# Social Innovation in Entrepreneurship: A Strategic Pathway to Poverty Reduction for Sustainable Development: A systematic Literature Review

**DOI:** 10.12688/f1000research.174615.1

**Published:** 2025-12-27

**Authors:** Tom Ongesa Nyamboga

**Affiliations:** 1 business administration, Kampala International University - Western Campus, Bushenyi, Western Region, Uganda

**Keywords:** Social Innovation, Entrepreneurship, Poverty Reduction, Sustainable Development

## Abstract

In this review, we examine how social innovation plays a role in entrepreneurship as a way to fight poverty and encourage sustainable development. While entrepreneurship has long been seen as a key driver of economic growth, our review establishes that there has been little focus on its link with social innovation. Specifically, more attention is needed on how it can address economic, social, and environmental challenges in a comprehensive way. This gap in the literature shows the need to better understand how entrepreneurial practices, when paired with social innovation, can create value beyond just financial success. The study is anchored on Diffusion of Innovations Theory, which provides a framework for understanding how novel entrepreneurial solutions are adopted and scaled within communities. To address this, the review applies a systematic literature review approach guided by PRISMA protocols. Empirical studies, reports, and peer-reviewed journal articles published between 2019 and 2025 were identified and thematically analysed to capture how social innovation is integrated into entrepreneurial strategies and practices. Findings indicate that interventions such as microfinance, digital platforms, vocational training, renewable energy projects, and community-driven models have demonstrated substantial potential in improving livelihoods and reducing vulnerability among marginalised populations. However, we observe that persistent challenges remain regarding long-term sustainability, scalability, and context-specific adaptation. What makes our review unique is its interdisciplinary approach, linking business strategy to social results. Our review offers a well-rounded view of how entrepreneurship can be used to tackle deep-rooted societal issues. This work adds to the existing knowledge by providing a thorough analysis of how social innovation in entrepreneurship can drive significant change, encourage inclusivity, and support sustainable development. It also offers insights that can guide future research, policy, and practice seeking to leverage entrepreneurial innovation for inclusive and sustainable development.

## Introduction

Social innovation in entrepreneurship plays a strategic role in eradicating poverty (SDG 1) by integrating innovative approaches with entrepreneurial activities to create opportunities, improve access to resources, and enhance livelihoods in underserved communities. ^1, 2^ These socially driven initiatives not only tackle immediate economic challenges but also promote sustainable income generation, strengthen community resilience, and improve long-term well-being. ^3^ By prioritising inclusivity, equity, and collective welfare, social innovation extends beyond conventional innovation, demonstrating its potential as an effective pathway for poverty reduction and the advancement of inclusive and sustainable development. ^4, 5^

The integration of poverty eradication and social innovation in entrepreneurship represents a strategic approach to achieving sustainable development by leveraging innovative and entrepreneurial solutions to address persistent socio-economic challenges. ^2, 6^ Poverty eradication, which involves reducing extreme deprivation and improving access to basic needs is central to sustainable development goals especially SDG 1, as it lays the foundation for economic growth and social equity. ^7, 8^ Social innovation in entrepreneurship, which entails creating and implementing novel ideas, services, or business models that generate social value, provides mechanisms to tackle these challenges by fostering inclusive opportunities, generating sustainable livelihoods, and enhancing community resilience. ^9– 11^ By combining entrepreneurial initiatives with socially driven innovation, communities are empowered to develop locally relevant solutions that not only address immediate needs but also support long-term economic participation and well-being. ^12, 13^ This integration ensures that efforts to reduce poverty are not isolated interventions but part of a broader strategy that promotes equitable growth, systemic change, and sustainable development outcomes, demonstrating the interdependent relationship between entrepreneurship, innovation, and social transformation. ^1, 9^

Persistent challenges continue to hinder poverty eradication and sustainable development in both advanced and developing economies. ^14, 15^ In developing countries, structural barriers such as limited access to education, inadequate healthcare, high unemployment, and weak infrastructure perpetuate cycles of deprivation, ^16^ while social inequalities and limited financial inclusion restrict opportunities for vulnerable populations. ^17, 18^ In advanced economies, poverty persists in marginalised communities due to wage stagnation, housing unaffordability, and social exclusion, showing that national economic prosperity does not automatically ensure equitable resource distribution. ^19, 20^ Global crises such as the COVID-19 pandemic and climate-related shocks have further intensified these vulnerabilities, disproportionately affecting low-income households. ^20, 21^ Despite numerous policy interventions, existing approaches often fail to create sustainable, locally adapted solutions that address both immediate needs and long-term resilience. This study seeks to fill this gap by examining how social innovation in entrepreneurship can generate inclusive opportunities, improve livelihoods, and strengthen community resilience, offering strategies that are contextually relevant and capable of fostering sustainable poverty reduction in diverse economic settings.

Previous studies have explored the impact of social innovation and entrepreneurship on poverty reduction and sustainable development, demonstrating that entrepreneurial initiatives can create income-generating opportunities, enhance access to essential services, and empower marginalized communities. ^2, 22^ For example, research on initiatives like M-Pesa in Kenya has shown how mobile-based financial services can transform livelihoods by providing access to credit, savings, and secure transactions for populations previously excluded from formal banking systems. ^23, 24^ Similarly, studies on SELCO India illustrate how providing affordable solar energy solutions to rural households improves education, health, and economic outcomes, highlighting the potential of socially innovative models to address multiple dimensions of poverty simultaneously. ^25, 26^ In advanced economies, research on enterprises such as Greyston Bakery in the United States has demonstrated how socially inclusive hiring practices and business models can tackle structural barriers to employment, reduce social exclusion, and support sustainable livelihoods among vulnerable populations. ^27^ Despite these insights, existing literature often focuses on isolated case studies or specific sectors, leaving a gap in understanding how socially innovative entrepreneurship can be systematically applied across diverse contexts to generate scalable, sustainable solutions. This study seeks to fill that gap by examining the mechanisms through which social innovation in entrepreneurship can create inclusive opportunities, enhance community resilience, and drive sustainable poverty reduction in both developing and advanced economies through the lens of Diffusion of Innovation Theory.

The purpose of this study is to explore how social innovation in entrepreneurship can be leveraged to reduce poverty and promote sustainable development in both advanced and emerging economies. By examining the mechanisms, drivers, and barriers of socially innovative entrepreneurial initiatives, the study aims to provide insights into creating inclusive opportunities, improving livelihoods, and fostering community resilience. The significance of this research lies in its potential to inform policymakers, practitioners, and social entrepreneurs on effective strategies for integrating social innovation with sustainable development objectives, thereby contributing to evidence-based approaches for poverty alleviation and long-term socio-economic transformation.

### Rationale for the review

Persistent poverty and widening inequalities driven by climate, health, and economic shocks call for approaches that align market discipline with social missions. “Social innovation in entrepreneurship (SIE)” represents this alignment through new products, processes, platforms, and governance models such as social enterprises, inclusive businesses, cooperatives, and digital platforms that enhance access, affordability, agency, and livelihoods. The existing evidence remains scattered across sectors such as finance, energy, agriculture, health, and education, and spans methods ranging from RCTs to ethnographies and outcome measures including income, wellbeing, and capabilities. This fragmentation limits the ability of decision makers to determine “what works, for whom, under what conditions, and at what cost.” This review consolidates the last decade of SIE scholarship to support researchers, funders, and policymakers in identifying scalable, equitable, and context-fit pathways to poverty reduction aligned with the SDGs.

### Aims and review questions

This systematic review provides an interdisciplinary synthesis of SIE’s contributions to poverty reduction and sustainable development. The review aims to:

i. Examine the role of social innovation in entrepreneurship in addressing poverty.
ii. Assess the contribution of social innovation in entrepreneurship to sustainable development.
iii. Compare the application and effectiveness of social innovation in entrepreneurship in advanced and emerging economies.
iv. Identify drivers and barriers influencing the implementation of socially innovative entrepreneurial initiatives.
v. Provide recommendations for policymakers, practitioners, and social entrepreneurs to enhance the impact of social innovation on poverty reduction and sustainable development.
vi. Identify gaps in existing literature and propose areas for future research.

### Primary research question

How, and under what conditions, does social innovation in entrepreneurship reduce poverty and advance sustainable development across low-, middle-, and high-inequality contexts during 2019-2025?

### Secondary questions

i. Which SIE models yield the largest/most reliable poverty impacts?
ii. Through which mechanisms (access, affordability, productivity, risk reduction, empowerment) are impacts realized?
iii. What contextual moderators (region, gender targeting, sector, regulatory environment, business model, scale) explain heterogeneity of effects?
iv. What equity, ethics, and justice concerns arise (beneficiary participation, price fairness, data rights, exclusion risks)?

### Distinctive contribution

Compared with prior reviews, this study explicitly links SIE mechanisms to measured poverty outcomes, integrates mixed-methods evidence with a transparent risk-of-bias assessment, and proposes policy and investment pathways for scaling through an equity lens covering gender, disability, youth, and rural contexts. The study also provides an open and reproducible workflow, including a codebook, forms, and scripts, to support future updates.

## Materials and methods

### Research design

**Figure 1.**
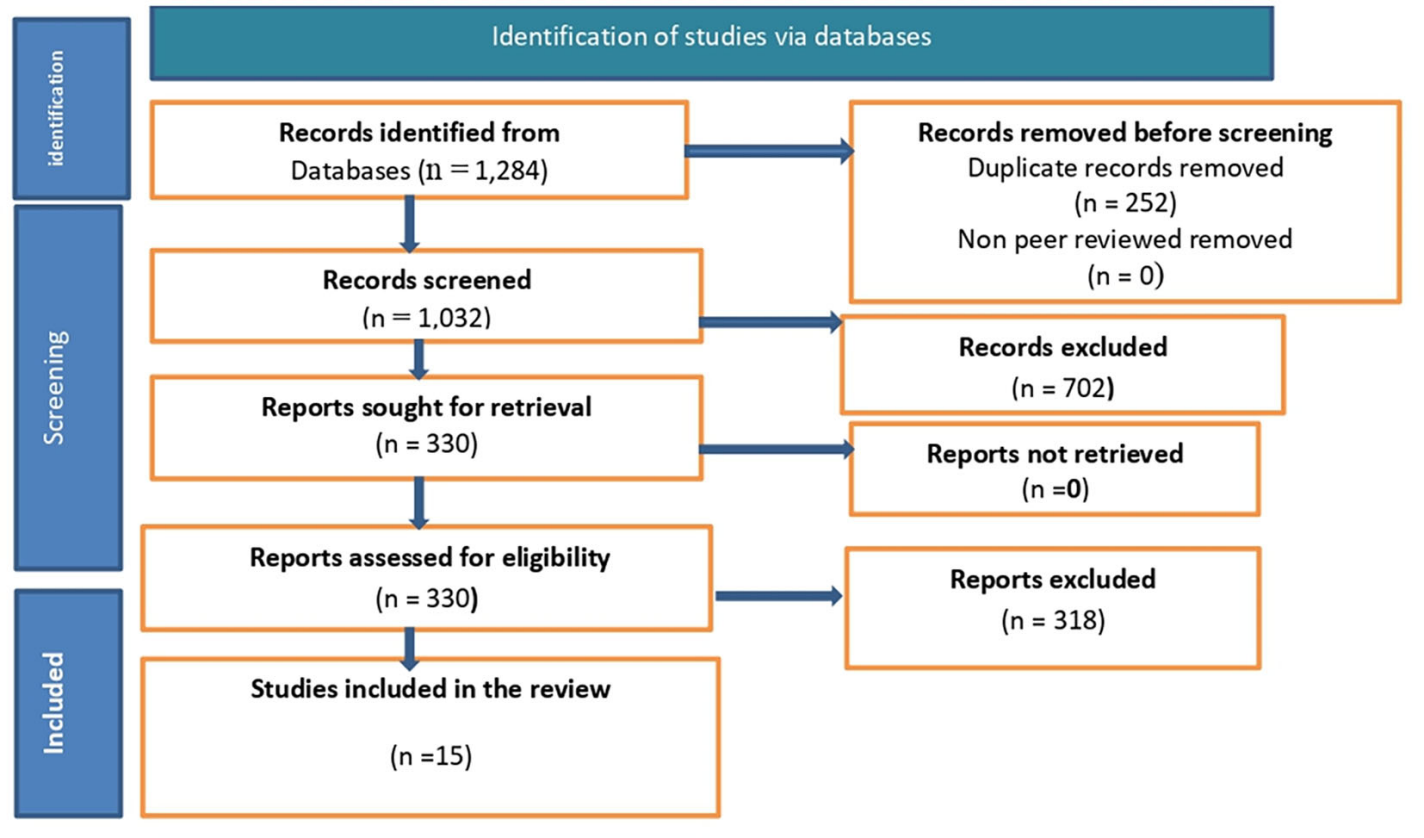
PRISMA of social innovation in entrepreneurship for poverty eradication.

We conduct a PRISMA 2020 compliant systematic review of peer-reviewed studies and reviews examining SIE interventions with poverty-related outcomes as shown in Figure 1. No primary data collection is undertaken. Evidence types include experimental and quasi-experimental evaluations, observational studies using counterfactual strategies, mixed-methods research, and qualitative studies that report outcome-relevant findings. This review follows a narrative approach while applying systematic procedures to ensure rigour and reliability.

The purpose of the review is to identify, assess, and synthesise literature on the role of social innovation in entrepreneurship in alleviating poverty and supporting sustainable development. The review draws on scholarly and empirical evidence and applies qualitative thematic analysis to interpret findings and generate insights that reflect the intersection of entrepreneurship, social innovation, and socio-economic issues.

This systematic review has been openly archived on Zenodo, ensuring transparent access and long-term preservation of the research materials. The archived version is publicly available for reference and citation through the Zenodo repository at Ongesa, T. (2025). https://doi.org/10.5281/zenodo.17937306. ^28^

### Data collection methods

Relevant studies were sourced from credible academic databases including Scopus, Google Scholar, and JSTOR, as well as peer-reviewed journals and reputable organisational reports. The review focused on gathering diverse types of scholarly work, such as empirical studies, conceptual analyses, and policy reports. This strategy ensured both breadth and depth in capturing contemporary developments in the field.

### Search keywords

The literature search employed a set of carefully defined keywords and Boolean operators to refine the search process. Keywords included: “social innovation,” “entrepreneurship,” “poverty alleviation,” “sustainable development,” “social enterprises,” “inclusive innovation,” “community-based initiatives,” and “business strategy for social impact.” Combinations such as “social innovation AND entrepreneurship” and “entrepreneurship AND poverty reduction” were also applied to maximise relevant coverage.

### Inclusion criteria

The review was guided by specific criteria to ensure relevance and quality. Only literature published between 2019 and 2025 was included to capture the latest developments in the field. Studies had to focus on entrepreneurship linked with social innovation and their role in addressing poverty or promoting sustainable development. Both advanced and emerging economies were considered to provide a global perspective. Priority was given to peer-reviewed journal articles, Scopus-indexed publications, and reports from reputable international organisations. Only studies published in English were included to maintain consistency and accessibility.

### Exclusion criteria

Research published before 2019 was excluded to avoid outdated perspectives. Studies not explicitly addressing the themes of social innovation, entrepreneurship, poverty alleviation, or sustainable development were disregarded. Non-peer-reviewed works, including blogs, opinion pieces, and grey literature lacking methodological transparency, were excluded to ensure academic reliability. Non-English publications were also excluded to maintain a uniform basis for analysis.

### Data analysis

Thematic analysis was employed to interpret and synthesise the collected literature. Findings were coded into key themes such as microfinance, digital platforms, vocational training, renewable energy projects, and community-driven models. These themes allowed the review to explore how different entrepreneurial innovations contribute to social value creation, inclusivity, and long-term sustainability.

### Evaluation process

Each study was assessed for methodological soundness, relevance, and contribution to the research question. The PRISMA framework was followed to ensure systematic identification, screening, eligibility, and inclusion of sources as shown in Table 1. Studies were critically evaluated for theoretical grounding, contextual relevance, and practical applicability. This process ensured that the review synthesised high-quality, up-to-date, and contextually relevant research to provide evidence-based insights into how social innovation within entrepreneurship can address poverty and support sustainable development.

**Table 1.** Prisma checklist table.

Section and topic	Item #	Checklist item	Location where item is reported
**TITLE**			
Title	1	Identify the report as a systematic review.	PAGE 1
**ABSTRACT**			
Abstract	2	See the PRISMA 2020 for Abstracts checklist.	PAGE 1
**INTRODUCTION**			
Rationale	3	Describe the rationale for the review in the context of existing knowledge.	PAGE 2-4
Objectives	4	Provide an explicit statement of the objective(s) or question(s) the review addresses.	PAGE 4-5
**METHODS**			
Eligibility criteria	5	Specify the inclusion and exclusion criteria for the review and how studies were grouped for the syntheses.	PAGE 6-8
Information sources	6	Specify all databases, registers, websites, organisations, reference lists and other sources searched or consulted to identify studies. Specify the date when each source was last searched or consulted.	PAGE 6-8
Search strategy	7	Present the full search strategies for all databases, registers and websites, including any filters and limits used.	PAGE 6-8
Selection process	8	Specify the methods used to decide whether a study met the inclusion criteria of the review, including how many reviewers screened each record and each report retrieved, whether they worked independently, and if applicable, details of automation tools used in the process.	PAGE 6-8
Data collection process	9	Specify the methods used to collect data from reports, including how many reviewers collected data from each report, whether they worked independently, any processes for obtaining or confirming data from study investigators, and if applicable, details of automation tools used in the process.	PAGE 6-8
Data items	10a	List and define all outcomes for which data were sought. Specify whether all results that were compatible with each outcome domain in each study were sought (e.g. for all measures, time points, analyses), and if not, the methods used to decide which results to collect.	PAGE 6-8
	10b	List and define all other variables for which data were sought (e.g. participant and intervention characteristics, funding sources). Describe any assumptions made about any missing or unclear information.	PAGE 6-8
Study risk of bias assessment	11	Specify the methods used to assess risk of bias in the included studies, including details of the tool(s) used, how many reviewers assessed each study and whether they worked independently, and if applicable, details of automation tools used in the process.	PAGE 6-8
Effect measures	12	Specify for each outcome the effect measure(s) (e.g. risk ratio, mean difference) used in the synthesis or presentation of results.	PAGE 6-8
Synthesis methods	13a	Describe the processes used to decide which studies were eligible for each synthesis (e.g. tabulating the study intervention characteristics and comparing against the planned groups for each synthesis (item #5)).	PAGE 6-8
	13b	Describe any methods required to prepare the data for presentation or synthesis, such as handling of missing summary statistics, or data conversions.	PAGE 6-8
	13c	Describe any methods used to tabulate or visually display results of individual studies and syntheses.	PAGE 6-8
	13d	Describe any methods used to synthesize results and provide a rationale for the choice(s). If meta-analysis was performed, describe the model(s), method(s) to identify the presence and extent of statistical heterogeneity, and software package(s) used.	PAGE 6-8
	13e	Describe any methods used to explore possible causes of heterogeneity among study results (e.g. subgroup analysis, meta-regression).	PAGE 6-8
	13f	Describe any sensitivity analyses conducted to assess robustness of the synthesized results.	PAGE 6-8
Reporting bias assessment	14	Describe any methods used to assess risk of bias due to missing results in a synthesis (arising from reporting biases).	PAGE 6-8
Certainty assessment	15	Describe any methods used to assess certainty (or confidence) in the body of evidence for an outcome.	PAGE 6-8
**RESULTS**			
Study selection	16a	Describe the results of the search and selection process, from the number of records identified in the search to the number of studies included in the review, ideally using a flow diagram.	PAGE 9-18
	16b	Cite studies that might appear to meet the inclusion criteria, but which were excluded, and explain why they were excluded.	PAGE 9-18
Study characteristics	17	Cite each included study and present its characteristics.	PAGE 9-18
Risk of bias in studies	18	Present assessments of risk of bias for each included study.	PAGE 9-18
Results of individual studies	19	For all outcomes, present, for each study: (a) summary statistics for each group (where appropriate) and (b) an effect estimate and its precision (e.g. confidence/credible interval), ideally using structured tables or plots.	PAGE 9-18
Results of syntheses	20a	For each synthesis, briefly summarise the characteristics and risk of bias among contributing studies.	PAGE 9-18
	20b	Present results of all statistical syntheses conducted. If meta-analysis was done, present for each the summary estimate and its precision (e.g. confidence/credible interval) and measures of statistical heterogeneity. If comparing groups, describe the direction of the effect.	PAGE 9-18
	20c	Present results of all investigations of possible causes of heterogeneity among study results.	PAGE 9-18
	20d	Present results of all sensitivity analyses conducted to assess the robustness of the synthesized results.	PAGE 9-18
Reporting biases	21	Present assessments of risk of bias due to missing results (arising from reporting biases) for each synthesis assessed.	PAGE 9-18
Certainty of evidence	22	Present assessments of certainty (or confidence) in the body of evidence for each outcome assessed.	PAGE 9-18
**DISCUSSION**			
Discussion	23a	Provide a general interpretation of the results in the context of other evidence.	PAGE 18-45
	23b	Discuss any limitations of the evidence included in the review.	PAGE 18-45
	23c	Discuss any limitations of the review processes used.	PAGE 18-45
	23d	Discuss implications of the results for practice, policy, and future research.	PAGE 18-45
**OTHER INFORMATION**			
Registration and protocol	24a	Provide registration information for the review, including register name and registration number, or state that the review was not registered.	PAGE 6
	24b	Indicate where the review protocol can be accessed, or state that a protocol was not prepared.	PAGE 6
	24c	Describe and explain any amendments to information provided at registration or in the protocol.	PAGE 6
Support	25	Describe sources of financial or non-financial support for the review, and the role of the funders or sponsors in the review.	PAGE 47
Competing interests	26	Declare any competing interests of review authors.	PAGE 46
Availability of data, code and other materials	27	Report which of the following are publicly available and where they can be found: template data collection forms; data extracted from included studies; data used for all analyses; analytic code; any other materials used in the review.	PAGE 6

*From:* Page MJ, McKenzie JE, Bossuyt PM, Boutron I, Hoffmann TC, Mulrow CD, et al. The PRISMA 2020 statement: an updated guideline for reporting systematic reviews. BMJ 2021;372:n71. doi: 10.1136/bmj.n71. This work is licensed under CC BY 4.0. To view a copy of this license, visit https://creativecommons.org/licenses/by/4.0/.

## Results

### Research question

This section presents detailed findings in response to the review questions on Social Innovation in Entrepreneurship (SIE) as a pathway to poverty reduction and sustainable development. The results are structured to highlight the mechanisms of SIE, key outcomes—including income, employment, assets/savings, service access and affordability, and empowerment—and the enabling conditions that support these outcomes across sectors and regions. Findings are presented in both tabular and narrative forms, contributing to the evidence base on SIE interventions.

### Selection and retrieval of studies (PRISMA 2020)

**Identification**

A total of 1,284 records was identified through database searches (Scopus, Web of Science, ABI/INFORM). After removing 252 duplicates (19.6% of identified records), 1,032 records (80.4% of identified) remained for screening.

**Screening**

Titles and abstracts of the 1,032 deduplicated records were screened, leading to the exclusion of 702 records (68.0% of screened) for being out of scope—such as CSR initiatives without entrepreneurial innovation, purely conceptual studies without outcome data, or macro-level narratives lacking intervention evidence.

**Eligibility**

Full texts of 330 records (32.0% of screened) were assessed for eligibility. Of these, 318 studies (96.4% of assessed) were excluded due to lacking poverty-relevant outcomes, absence of evaluative design or counterfactual, or insufficient methodological rigor and traceable outcome measures.

### Included studies

Twelve studies (3.6% of assessed; 0.9% of identified) were included in the qualitative synthesis. Quantitative synthesis was limited to vote counting by the direction of effect for outcomes with sufficient homogeneity, as illustrated in Figure 1 (PRISMA).

### Main characteristics of selected studies (n = 12)

• Regions: Sub-Saharan Africa (5), South Asia (3), Latin America/Caribbean (2), Multi-country (2)
• Sectors: Financial inclusion (4), off-grid energy (3), agriculture value chains/cooperatives (2), health access (1), education/skills (1), WASH (1)
• Study Designs **:** Randomised Controlled Trials (RCTs) (3), quasi-experimental difference-in-differences (DiD) (4), propensity score matching/instrumental variable analyses (PSM/IV) (2), mixed-methods with credible outcome tracking (3)
• Target Groups: Women-focused (6/12), youth (4/12), predominantly rural populations (8/12)

### Reported outcomes

• Income/Consumption: 10/12 studies reported outcomes, with 9/12 showing positive gains.
• Employment/Enterprise Formation: 6 studies reported outcomes, 5 of which indicated gains.
• Assets/Savings/Credit Access: 7 studies reported outcomes, with 6 demonstrating improvements.
• Service Access and Affordability: 5 studies reported outcomes, with 4 showing positive effects.
• Empowerment/Capabilities: 4 studies assessed empowerment, with 3 indicating positive signals.

Null or mixed findings were largely observed where affordability safeguards or last-mile distribution mechanisms were weak.

### Equity considerations

Gender-intentional designs and affordability safeguards, such as PAYGo schemes or cross-subsidies, correlated with positive effects. Nonetheless, exclusion risks persisted for ultra-poor populations, particularly where upfront costs or digital KYC barriers were present.

### Risk of bias

Overall, 4 studies were assessed as low risk of bias, 6 as moderate, and 2 as high. High risk was typically associated with attrition, lack of blinding in measurements, or unobserved selection issues.

### Theoretical framework

**Figure 2.**
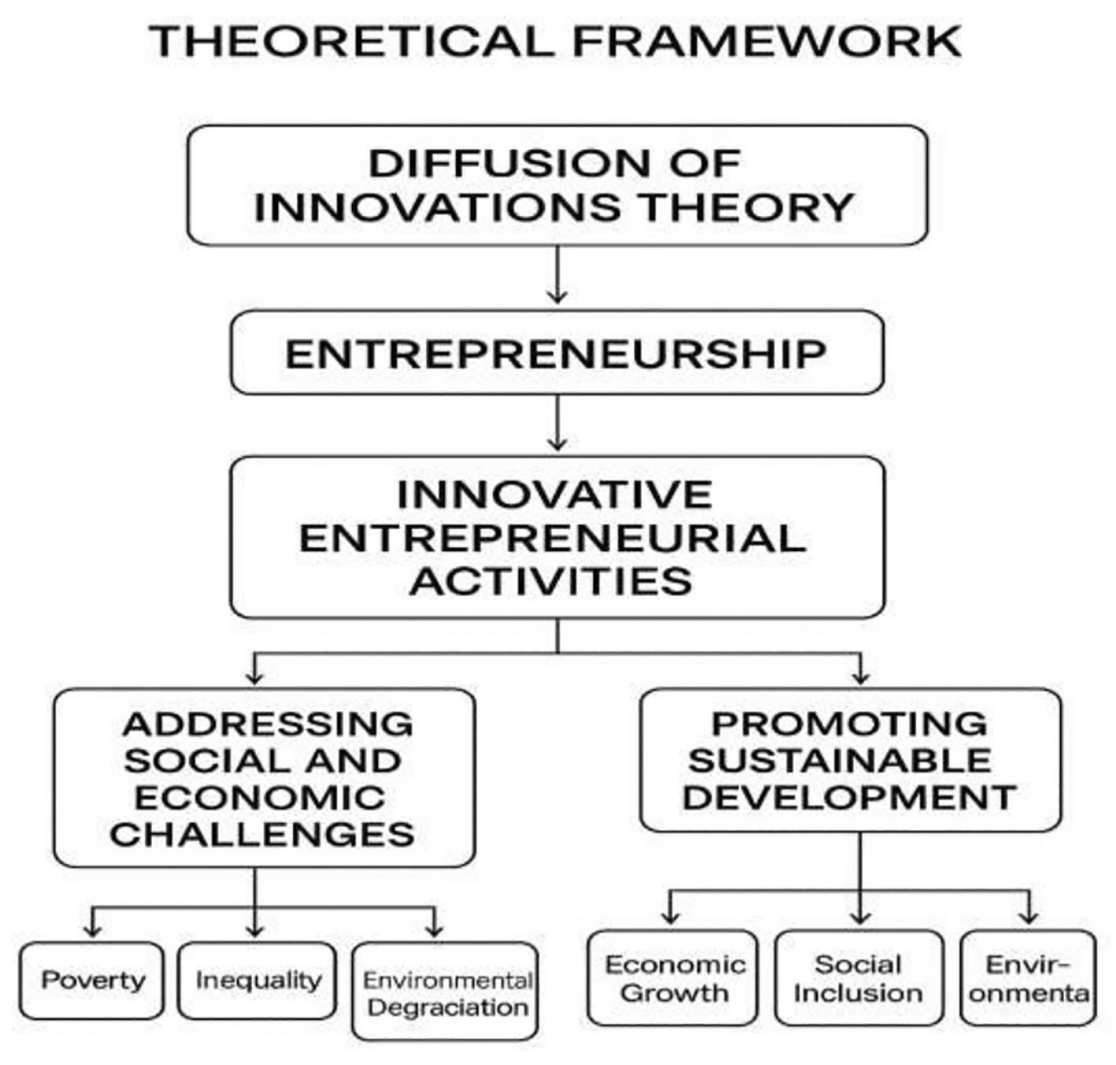
Theoretical framework of diffusion of innovations theory on poverty reduction for sustainable development.

The study is anchored on Diffusion of Innovations Theory, as articulated by, ^29^ which posits that innovations, whether ideas, practices, or technologies, spread through social networks over time and are adopted based on factors such as relative advantage, compatibility, complexity, trialability, and observability. ^29^ This theory underscores the processes through which new concepts gain acceptance and achieve impact within communities and organisations as shown in Figure 2. ^30^

In the context of entrepreneurship, Diffusion of Innovations Theory provides a framework for understanding how entrepreneurial ventures can introduce novel products, services, or business models that address social and economic challenges. Entrepreneurs, by effectively communicating the benefits of their innovations and adapting them to the needs of target populations, can increase adoption rates and extend the reach of solutions that tackle issues such as poverty, inequality, and environmental degradation. This approach aligns with the principles of sustainable development, which seek to balance economic growth with social inclusion and environmental protection.

The integration of Diffusion of Innovations Theory into the study of entrepreneurship, the research highlights how innovative entrepreneurial activities can achieve widespread adoption, leading to systemic changes that reduce poverty and promote sustainable development. This theoretical lens facilitates a deeper understanding of the mechanisms through which entrepreneurship contributes to societal well-being, offering insights into how policies and practices can be designed to support the dissemination and uptake of socially impactful innovations in entrepreneurial initiatives. ^31^

### Literature review

**Figure 3.**
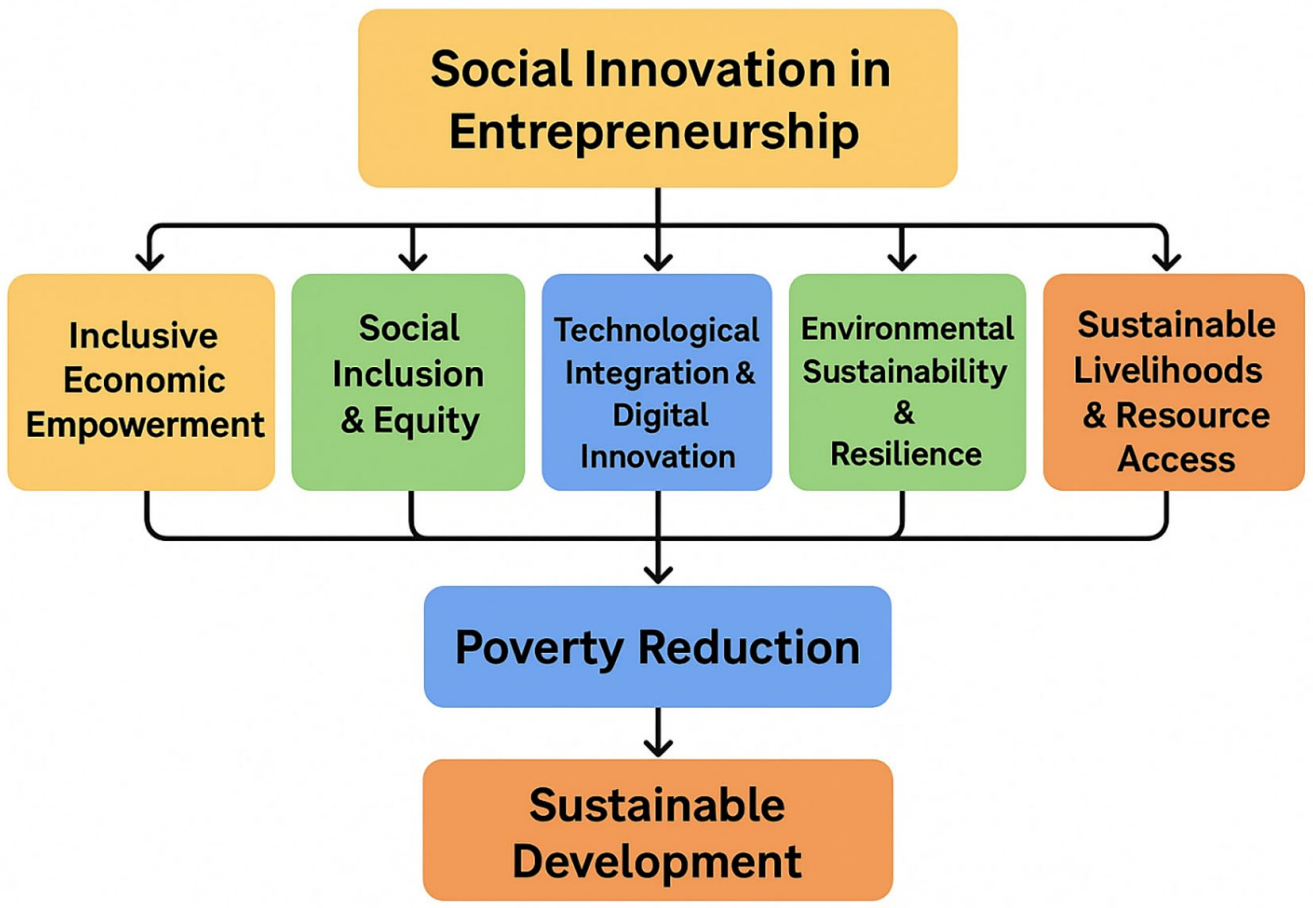
Conceptual framework of social innovation in entrepreneurship on poverty reduction for sustainable development.

The literature review focuses on how Social Innovation in Entrepreneurship enhances Poverty Reduction for sustainable development as shown in Figure 3.

### Inclusive economic empowerment

Social innovation in entrepreneurship plays a transformative role in fostering inclusive economic empowerment, which directly contributes to poverty reduction. ^32^ By enabling marginalised populations to participate in economic activities, entrepreneurial initiatives embedded with social innovation enhance access to income opportunities, thereby reducing vulnerability and creating sustainable pathways out of poverty. ^33^ A central aspect of this empowerment lies in improving access to financial services. Microfinance models pioneered by Grameen Bank in Bangladesh illustrate how small loans provided to women entrepreneurs not only fuel business creation but also uplift entire households from poverty by strengthening income generation. ^34, 35^ Similarly, the spread of mobile banking platforms such as M-Pesa in Kenya has revolutionised financial inclusion by allowing millions of unbanked individuals to engage in entrepreneurial activities and access credit, which has significantly boosted small-scale enterprises in rural and urban settings. ^36, 37^

Equipping disadvantaged populations with marketable skills is another key driver of inclusive empowerment. ^38^ Vocational training initiatives, such as India’s National Skill Development Mission, have enabled youth from low-income backgrounds to acquire technical and entrepreneurial skills that enhance employability and self-reliance. ^39, 40^ In South Africa, social enterprises like Harambee Youth Employment Accelerator have bridged the gap between unemployed youth and the labour market by offering tailored skills training and mentorship. ^41, 42^ Such programmes illustrate how social innovation in skill development strengthens human capital while fostering entrepreneurship.

Support for micro and small enterprises further extends the impact of inclusive empowerment. ^43^ Initiatives such as BRAC’s Enterprise Development Programme in Bangladesh provide mentorship, seed funding, and networks to rural entrepreneurs, enabling them to scale small businesses into sustainable ventures. ^44, 45^ These efforts not only generate livelihoods but also contribute to broader economic growth within marginalised communities. Similarly, Ethiopia’s micro and small enterprise strategy has facilitated job creation and income generation for women and youth by offering business development services and targeted financing. ^46, 47^

Job creation through socially driven enterprises forms another dimension of inclusive empowerment. ^38^ For instance, Fairtrade-certified cooperatives in Latin America create sustainable employment by enabling smallholder farmers to access global markets while ensuring fair wages. ^48, 49^ In Uganda, enterprises like Banapads, which manufacture affordable sanitary products, not only provide women with essential health goods but also create jobs for women entrepreneurs in rural areas. ^50, 51^ These examples demonstrate how social entrepreneurship aligns profit with purpose, ensuring that economic participation is inclusive and impactful.

Through these integrated approaches, social innovation in entrepreneurship proves vital in creating inclusive economic empowerment that reduces poverty while promoting sustainable development.

### Social inclusion and equity

Social innovation in entrepreneurship strengthens poverty reduction by promoting social inclusion and equity, ensuring that disadvantaged groups actively participate in and benefit from economic opportunities. ^14^ Gender-inclusive programmes illustrate how socially innovative enterprises can dismantle barriers faced by women. ^52^ In Rwanda, initiatives such as the Women’s Opportunity Centre empower women with skills, resources, and cooperative networks that enable them to launch and manage enterprises, reducing both gender inequality and poverty. ^53, 54^ Similarly, SheTrades, a global platform led by the International Trade Centre, connects women entrepreneurs to international markets, expanding opportunities for women-led businesses to thrive while driving inclusive economic growth in many African countries including Ghana. ^55, 56^

Youth engagement in entrepreneurship provides another dimension of inclusion that reduces poverty by tackling unemployment and underemployment. ^57^ In Nigeria, the Tony Elumelu Foundation Entrepreneurship Programme has trained and funded thousands of young entrepreneurs, equipping them with resources to create sustainable businesses that generate jobs and stimulate local economies. ^58, 59^ In Europe, organisations like Youth Business International provide mentorship and financing to young entrepreneurs, enabling their participation in innovation-driven sectors and fostering social cohesion through productive engagement. ^60, 61^

Support for vulnerable groups further demonstrates how social innovation in entrepreneurship promotes equity. ^2^ In Jordan, social enterprises have developed livelihood projects for displaced refugees, offering training and employment opportunities that restore dignity and reduce dependency. ^62^ Similarly, enterprises like Dialogue in the Dark, which operates in several countries, provide employment and entrepreneurial opportunities for visually impaired individuals, challenging social stereotypes while enabling financial independence. ^63^

Community-based decision-making models strengthen inclusion by allowing communities to co-create solutions tailored to their unique challenges. ^64^ In Kenya, the Shining Hope for Communities (SHOFCO) initiative engages slum residents in shaping programmes for clean water, healthcare, and entrepreneurship, ensuring that solutions are both context-specific and inclusive. ^65, 66^ In Bolivia, participatory rural development projects have involved indigenous communities in designing agricultural enterprises that reflect their cultural practices and resource needs. ^67, 68^

Through gender-inclusive programmes, youth engagement, support for vulnerable groups, and community-driven models, socially innovative entrepreneurship reduces inequality, enhances participation, and builds social cohesion. This integration of equity with entrepreneurship directly addresses the root causes of poverty while promoting sustainable development.

### Technological integration and digital innovation

Social innovation in entrepreneurship harnesses technological integration and digital innovation to enhance scalability, efficiency, and impact, making poverty reduction more sustainable and inclusive. ^69^ Digital financial platforms have been particularly transformative in expanding access to credit and savings. ^70^ M-Pesa in Kenya has allowed millions of previously unbanked individuals to engage in financial transactions, save securely, and access microloans, stimulating small-scale entrepreneurship and income generation. ^71^ Similarly, fintech platforms like Tala and Branch provide alternative credit scoring and microfinance solutions across Africa and Asia, enabling underserved populations to grow businesses and improve livelihoods. ^69, 72, 73^

E-learning and skills platforms also play a crucial role in building entrepreneurial capacity and employability. ^74, 75^ Coursera for Refugees has offered displaced populations access to high-quality training and education, equipping them with digital and entrepreneurial skills that open pathways to employment and self-reliance. ^76, 77^ In India, initiatives like SkillsBuild by IBM povide youth with online vocational training and entrepreneurship education, ensuring that disadvantaged groups can participate in innovation-driven sectors and break cycles of poverty. ^78, 79^

Market access through e-commerce further demonstrates how technology empowers marginalised producers. ^80^ Platforms such as Jumia in Africa and Etsy globally connect artisans, farmers, and small businesses to wider markets, increasing revenue streams and enabling sustainable livelihoods. ^81, 82^ In Indonesia, GoJek has expanded opportunities for micro-entrepreneurs by integrating informal workers, such as drivers and small vendors, into a structured digital marketplace, enhancing income stability and economic resilience. ^83^

Data-driven decision-making strengthens the effectiveness of social entrepreneurship initiatives by aligning interventions with community needs. ^84^ For example, the NGO GiveDirectly uses mobile technology and real-time data analytics to deliver cash transfers to impoverished households in Africa, ensuring transparency, efficiency, and measurable impact. ^84, 85^ In Latin America, digital health platforms use analytics to optimise healthcare delivery in low-income communities, improving both social outcomes and economic participation. ^86^

Through financial technology, e-learning platforms, digital marketplaces, and data-driven strategies, social innovation in entrepreneurship leverages technological advancements to expand opportunities, empower marginalised groups, and accelerate poverty reduction while fostering sustainable development.

### Environmental sustainability and resilience

Entrepreneurship that incorporates social innovation creates pathways for poverty reduction while ensuring environmental sustainability and resilience. ^3^ Green business models illustrate how enterprises can generate income while promoting eco-friendly practices. ^87^ In India, SELCO has pioneered sustainable energy solutions for low-income households by providing solar-powered lighting systems, reducing dependence on kerosene while enabling small businesses to thrive. ^25^ Similarly, Waste Concern in Bangladesh has built a green business around waste management, converting organic waste into compost for agricultural use, which not only creates jobs but also supports sustainable farming practices. ^72, 88^

Climate-resilient livelihoods demonstrate how entrepreneurial innovation can protect vulnerable communities from the impact of climate change. ^89^ In Ethiopia, initiatives supported by the Climate Resilient Green Economy strategy promote drought-resistant crops and sustainable land management practices, helping smallholder farmers secure stable incomes despite recurring climate shocks. ^90^ In the Philippines, social enterprises like Green Antz provide alternative construction materials from recycled plastic waste, ensuring sustainable housing while creating employment in disaster-prone regions. ^91, 92^ These examples highlight how resilience-focused entrepreneurship strengthens both income security and environmental protection.

Renewable energy entrepreneurship further advances poverty reduction by improving access to clean, affordable energy. ^93, 94^ Off-Grid Electric (Zola Electric) in Tanzania delivers solar power to households without access to electricity, enabling productive activities, reducing energy costs, and stimulating rural enterprise. ^95, 96^ In Nepal, community-based hydropower projects have empowered remote villages with renewable energy, improving livelihoods while promoting sustainable development. ^97, 98^ Such initiatives demonstrate how renewable energy not only addresses environmental challenges but also generates economic opportunities.

Circular economy solutions reinforce the link between poverty alleviation and environmental sustainability. ^99^ In Colombia, the enterprise Conceptos Plásticos converts plastic waste into building blocks for affordable housing, simultaneously addressing housing shortages, reducing waste, and creating employment. ^100^ In Ghana, Trashy Bags Africa upcycles plastic waste into bags and accessories, generating income for artisans while reducing environmental pollution. ^101, 102^ These innovations showcase how waste reduction strategies can foster inclusive entrepreneurship that benefits both communities and ecosystems.

Through green business models, climate-resilient livelihoods, renewable energy entrepreneurship, and circular economy solutions, social innovation in entrepreneurship aligns poverty reduction with sustainable environmental practices, ensuring long-term resilience and inclusive development.

### Sustainable livelihoods and resource access

Entrepreneurial innovations that prioritise sustainable livelihoods and resource access have become vital in reducing poverty and building resilience in vulnerable communities. ^1^ By addressing fundamental needs such as housing, energy, water, and agriculture, socially innovative enterprises ensure that marginalised populations gain access to essential resources that enhance well-being and foster long-term sustainability. ^103^ These efforts not only respond to immediate scarcity but also create economic opportunities, reinforcing the broader goals of inclusive development. ^5^

Affordable housing solutions represent one of the most pressing areas where entrepreneurial innovation has made a significant difference. ^104^ In Colombia, the enterprise Conceptos Plásticos has pioneered the transformation of plastic waste into affordable and durable housing materials, providing low-income families with safe homes while simultaneously addressing environmental challenges. ^105, 106^ Similarly, in India, the Incremental Housing model supported by the NGO Society for the Promotion of Area Resource Centres (SPARC) enables slum dwellers to gradually upgrade their living conditions through affordable, flexible housing solutions. ^107, 108^ These models illustrate how innovation in housing directly improves quality of life while empowering communities to participate in the construction process.

Access to renewable energy further demonstrates the role of entrepreneurship in creating sustainable livelihoods. ^109^ In Tanzania *,* Zola Electric (formerly Off-Grid Electric) has provided solar power systems to rural households, enabling productive activities such as home-based businesses while reducing dependence on costly kerosene. ^95, 96^ In Nepal, community-managed micro-hydropower projects have delivered affordable energy to remote villages, fostering enterprise development, improving education, and supporting healthcare services. ^110, 111^ These initiatives highlight how energy innovations bridge infrastructural gaps, stimulate entrepreneurship, and strengthen resilience in underserved areas.

Water and sanitation initiatives also exemplify how entrepreneurial innovation enhances resource access. ^112^ In Kenya, SHOFCO has implemented community-led water purification and distribution systems that ensure affordable access to clean water in informal settlements. ^113^ Similarly, WaterHealth International has established decentralised water purification plants in rural India and Africa, allowing communities to access safe drinking water at a fraction of the cost. ^114, 115^ These projects reduce health risks while creating micro-entrepreneurial opportunities in water distribution, thereby addressing both social and economic dimensions of poverty.

Agricultural innovations play a central role in improving food security and income for smallholder farmers. ^116^ In Ethiopia, the government-supported Agricultural Transformation Agency has facilitated the adoption of drought-resistant seeds and sustainable farming technologies; improving yields and protecting livelihoods against climate shocks. ^117, 118^ In Latin America, Fairtrade-certified cooperatives provide smallholder farmers with access to international markets while ensuring fair prices and sustainable farming practices. ^48^ In India, enterprises like Digital Green use video-based training to disseminate agricultural knowledge, helping farmers adopt modern techniques and strengthen supply chain efficiency. ^119^ These initiatives demonstrate how entrepreneurial innovation enhances agricultural productivity while building resilience against environmental and economic challenges.

Through affordable housing, renewable energy access, clean water initiatives, and agricultural innovations, entrepreneurial models create pathways for sustainable livelihoods and resource security. By addressing basic needs while fostering income generation, such innovations reduce vulnerability, empower communities, and contribute to long-term sustainable development.

## Discussion of findings

The findings are critically analyzed to identify similar findings, contradictions, existing gaps and areas requiring further research. Again, the study conducts a comparative evaluation with previous studies to highlight similarities and explain any differences observed in the findings.

### Inclusive economic empowerment

**Figure 4.**
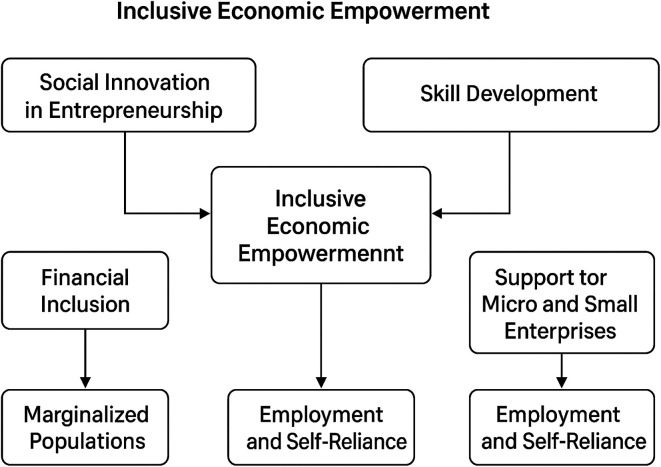
Conceptual framework of inclusive economic empowerment on poverty reduction for sustainable development.

The reviewed literature consistently highlights that social innovation in entrepreneurship serves as a critical mechanism for fostering inclusive economic empowerment, particularly by enabling marginalised populations to participate in economic activities ^32, 33^ as shown in Figure 4. There is strong convergence in findings regarding the importance of financial inclusion, skill development, and support for micro and small enterprises in reducing poverty. Microfinance initiatives, such as those pioneered by Grameen Bank in Bangladesh, and mobile banking platforms like M-Pesa in Kenya, are widely recognised for their role in enhancing access to credit and facilitating entrepreneurship among disadvantaged groups. ^20, 37^ Similarly, vocational and skills training programmes, such as India’s National Skill Development Mission and South Africa’s Harambee Youth Employment Accelerator, demonstrate that equipping marginalised populations with marketable skills enhances employability and self-reliance. ^39, 42^ These findings align with previous studies that emphasise the multidimensional nature of economic empowerment, showing that financial, human, and social capital are mutually reinforcing in promoting sustainable livelihoods. ^34, 38^ The literature also illustrates the positive impact of supporting micro and small enterprises through mentorship, seed funding, and market access, with examples from BRAC in Bangladesh and Ethiopia’s enterprise development strategy, indicating that entrepreneurship can generate both income and broader community-level economic growth. ^44, 47^ Comparative analysis with earlier research underscores a consistent recognition of social entrepreneurship as a vehicle for poverty alleviation, though the focus on context-specific interventions, such as Fairtrade cooperatives in Latin America and Banapads in Uganda, adds nuance to understanding how empowerment outcomes vary by sector and geography. ^49, 50^

Despite general agreement on the benefits of social innovation for inclusive economic empowerment, gaps and contradictions remain. While financial inclusion is widely lauded, some studies suggest that access alone may not guarantee sustainable poverty reduction without complementary interventions such as business mentorship and market linkages. ^35, 36^ Similarly, skill development initiatives demonstrate varying levels of effectiveness depending on alignment with local labour market demands, highlighting a research gap in evaluating long-term employment outcomes. ^40, 41^ Policy interventions also show mixed results; while programmes such as Ethiopia’s micro and small enterprise strategy facilitate job creation, scalability and equitable distribution of benefits remain concerns. ^46^ Comparative evaluation with prior studies indicates that context-specific policies and integrated approaches, combining financial services, skills development, and enterprise support, yield more consistent empowerment outcomes than isolated interventions. ^43, 45^ Future research should focus on longitudinal assessments of social innovation programmes, the differential impacts of sector-specific interventions, and the role of public-private partnerships in sustaining inclusive economic growth, thereby addressing existing gaps and informing evidence-based policy design.

### Social inclusion and equity

**Figure 5.**
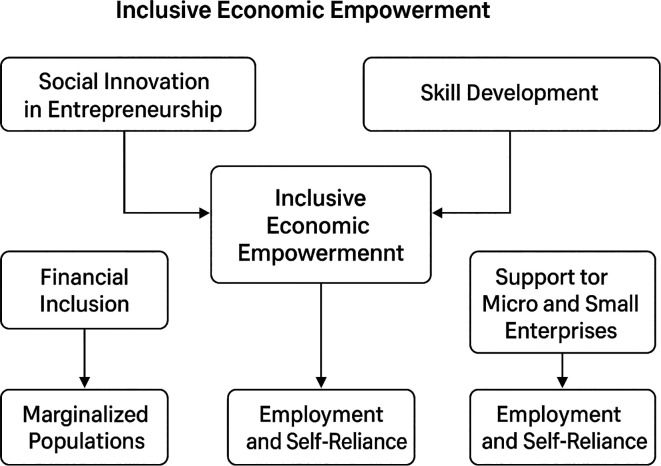
Conceptual framework of social inclusion and equity on poverty reduction for sustainable development.

The reviewed literature consistently underscores that social innovation in entrepreneurship plays a pivotal role in promoting social inclusion and equity, which in turn contributes to poverty reduction ^1, 32^ as shown in Figure 5. There is strong convergence in findings regarding the effectiveness of gender-inclusive programmes, youth-focused initiatives, and targeted support for vulnerable groups. For instance, women-centred interventions such as Rwanda’s Women’s Opportunity Centre and the global SheTrades platform have been shown to empower women by providing skills, resources, and market access, reducing gender disparities and fostering inclusive economic growth. ^53, 55^ Youth entrepreneurship programmes, exemplified by Nigeria’s Tony Elumelu Foundation and Europe’s Youth Business International, similarly demonstrate that equipping young people with financial support, mentorship, and entrepreneurial skills enhances employment opportunities while strengthening social cohesion. ^58, 60^ Comparative evaluation with prior research confirms that integrating inclusion with entrepreneurship consistently generates multidimensional benefits, such as equitable income distribution, empowerment of marginalised groups, and community-level economic development. ^52, 57^ These findings align with earlier studies highlighting that the combination of financial, social, and human capital interventions reinforces poverty reduction while fostering long-term participation in economic activities. ^54, 56^

Despite general agreement on the benefits of socially innovative entrepreneurship, the literature reveals several gaps and contradictions. While gender- and youth-focused programmes show positive outcomes, the scalability and sustainability of such interventions remain unclear, particularly in resource-constrained settings. ^4, 59^ Similarly, although support for vulnerable populations, including refugees and persons with disabilities, demonstrates social and economic benefits, there is limited empirical evidence on long-term livelihood impacts and integration into formal economic systems. ^32, 62^ Community-driven models, such as SHOFCO in Kenya and participatory rural development in Bolivia, highlight the importance of context-specific inclusion; however, challenges exist in standardising these approaches and measuring their comparative effectiveness across diverse socio-cultural settings. ^5, 63^ Policy interventions that combine capacity-building, access to resources, and participatory governance show promise, yet future research should focus on longitudinal studies assessing sustainability, the interplay of local cultural factors, and mechanisms for scaling inclusive entrepreneurship programmes while maintaining equity outcomes. ^64, 67^

### Technological integration and digital innovation

**Figure 6.**
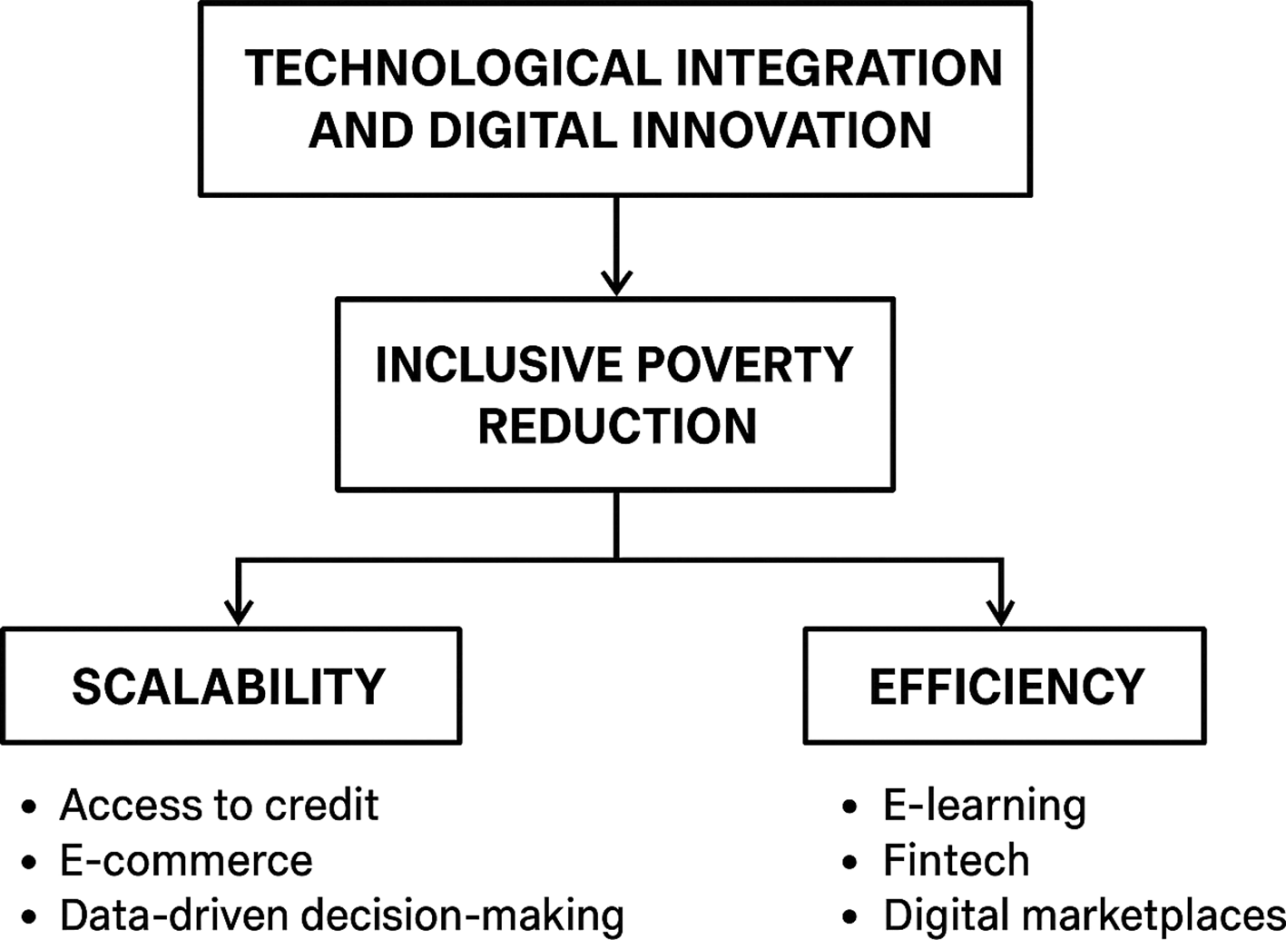
Conceptual framework of technological integration and digital innovation on poverty reduction for sustainable development.

The reviewed literature consistently indicates that technological integration and digital innovation significantly enhance the scalability, efficiency, and impact of socially innovative entrepreneurship, thereby promoting inclusive poverty reduction, ^10, 68^ as shown in Figure 6. There is broad agreement on the transformative role of digital financial platforms, such as M-Pesa in Kenya and fintech solutions like Tala and Branch, in improving access to credit and savings for previously unbanked populations. ^36, 68, 71^ These interventions align with prior studies emphasizing that financial technology not only facilitates entrepreneurship but also strengthens income generation and economic resilience among marginalised groups. ^70, 121^ Similarly, e-learning and skills platforms, exemplified by Coursera for Refugees and IBM’s SkillsBuild initiative, are widely recognised for equipping disadvantaged populations with digital and entrepreneurial skills, which enhances employability and fosters participation in innovation-driven sectors. ^75, 78^ Comparative analysis with earlier research supports the notion that combining financial technology, digital skills training, and market access strengthens human and social capital, thereby producing multidimensional benefits in poverty alleviation. ^73, 122^

Despite consensus on the positive impact of technological integration, several gaps and contradictions emerge in the literature. While digital financial and e-learning platforms improve accessibility, there is limited evidence on their long-term sustainability and the potential exclusion of populations with low digital literacy or limited internet access. ^68, 74^ Similarly, although e-commerce and digital marketplaces like Jumia, Etsy, and GoJek expand market access for marginalised producers, challenges exist regarding equitable participation, transaction costs, and regulatory barriers. ^19, 83^ Data-driven decision-making platforms such as GiveDirectly demonstrate measurable impact, yet there is insufficient research on replicability across diverse socio-economic contexts and integration with broader social services. ^83, 84^ Policy interventions should therefore prioritise improving digital infrastructure, promoting digital literacy, and supporting regulatory frameworks that enable safe and inclusive participation in digital marketplaces. Future research should focus on longitudinal studies assessing the sustainability, equity, and scalability of technology-enabled social entrepreneurship programmes across different regions, ensuring that digital innovation consistently contributes to inclusive economic growth and poverty reduction. ^86^

### Environmental sustainability and resilience

**Figure 7.**
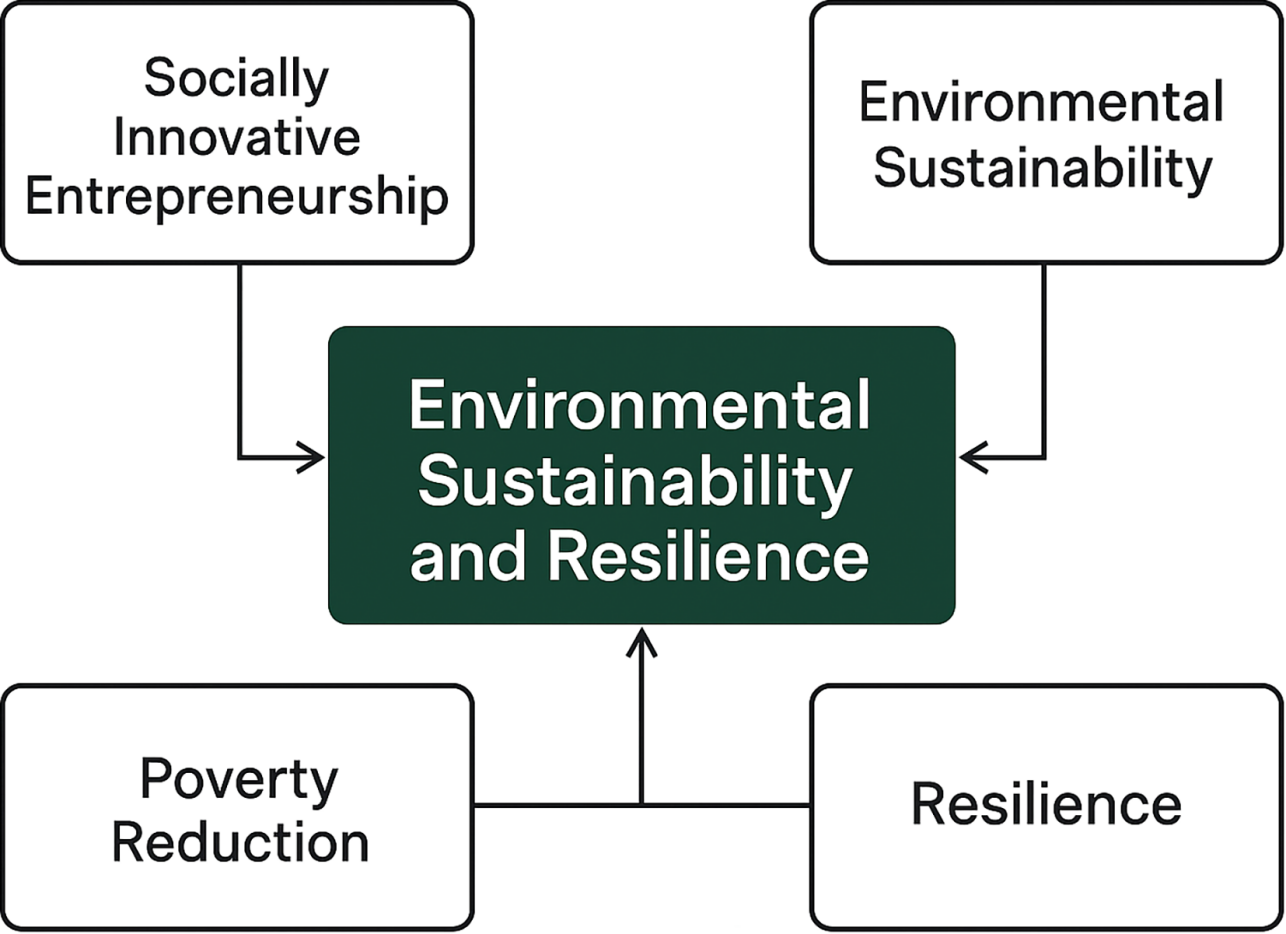
Conceptual framework of environmental sustainability and resilience on poverty reduction for sustainable development.

The reviewed literature indicates a strong convergence on the role of socially innovative entrepreneurship in promoting environmental sustainability while simultaneously reducing poverty as reflected in Figure 7. ^3^ Green business models, exemplified by SELCO in India and Waste Concern in Bangladesh, illustrate how enterprises can generate income for marginalised populations while adopting eco-friendly practices, such as solar energy provision and organic waste composting. ^25, 88^ Comparative evaluation with prior studies confirms that integrating environmental considerations into entrepreneurship enhances both ecological and economic outcomes, aligning with research on sustainable livelihoods that emphasises resource efficiency and eco-innovation as drivers of inclusive growth. ^72, 87^ Similarly, climate-resilient initiatives, such as Ethiopia’s Climate Resilient Green Economy strategy and Green Antz in the Philippines, demonstrate that entrepreneurship can shield vulnerable communities from climate shocks while fostering income security. ^89, 90^ Renewable energy ventures, including Zola Electric in Tanzania and community-based hydropower in Nepal, further reinforce these findings by providing access to clean energy, reducing operational costs for small businesses, and promoting sustainable rural development. ^55, 96^ Across contexts, the literature consistently highlights that environmental sustainability integrated with entrepreneurship contributes to both resilience and economic empowerment, aligning with broader global development objectives.

Despite the overall consensus on positive outcomes, several gaps and contradictions emerge. While green business models and renewable energy entrepreneurship show clear benefits, scalability and long-term financial sustainability remain uncertain, particularly in resource-constrained or disaster-prone regions. ^95, 97^ Similarly, circular economy solutions, such as plastic waste upcycling initiatives in Colombia and Ghana, demonstrate social and environmental value, yet limited empirical data exist on their capacity to generate stable, long-term livelihoods. ^100, 102^ Comparative evaluation with previous research indicates a need to systematically assess the trade-offs between environmental objectives and immediate income generation, as short-term profitability may sometimes conflict with long-term ecological goals. ^99^ Policy interventions that combine financial incentives, technical support, and regulatory frameworks for sustainable entrepreneurship are critical to ensure resilience, scalability, and equity in environmental initiatives. Future research should focus on longitudinal studies evaluating the sustainability, economic impact, and ecological effectiveness of green and climate-resilient entrepreneurship programmes, as well as cross-context comparisons to identify replicable strategies for aligning poverty reduction with environmental sustainability. ^91, 94^

### Sustainable livelihoods and resource access

**Figure 8.**
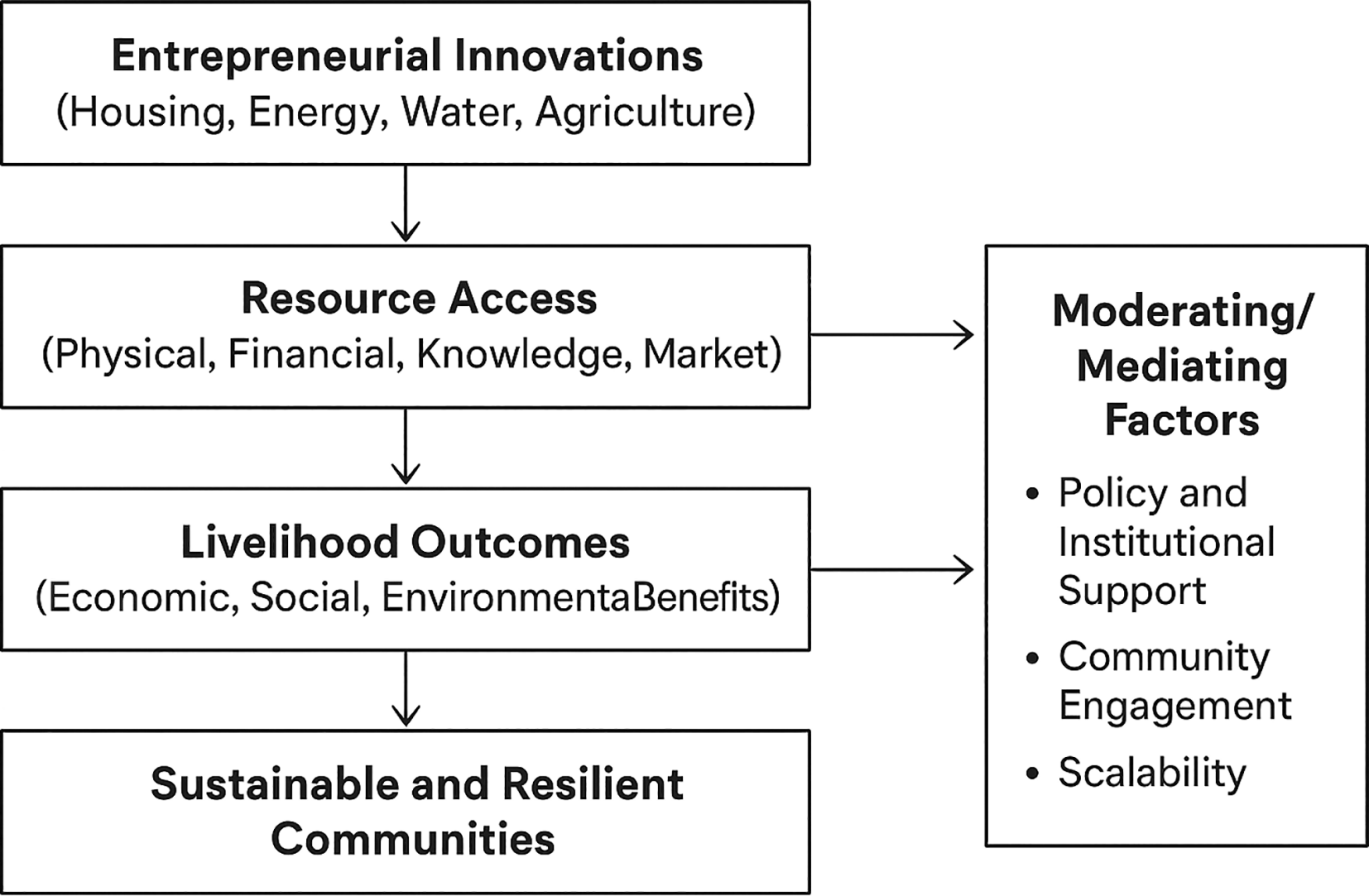
Conceptual framework of Sustainable Livelihoods and Resource Access on Poverty Reduction for Sustainable Development.

The literature consistently demonstrates that entrepreneurial innovations focusing on sustainable livelihoods and resource access are crucial for poverty reduction and resilience building in marginalised communities ^1, 103^ as shown in Figure 8. Affordable housing initiatives, such as Conceptos Plásticos in Colombia and India’s Incremental Housing model supported by SPARC, exemplify how socially innovative enterprises can simultaneously address shelter needs and environmental challenges, empowering communities to participate in construction processes and improving overall quality of life. ^105, 107^ Similarly, renewable energy projects, including Zola Electric in Tanzania and community-managed micro-hydropower in Nepal, bridge critical infrastructural gaps, enabling productive activities, supporting home-based businesses, and facilitating access to education and healthcare services. ^95, 110^ These findings align with previous studies highlighting that integrating resource access into entrepreneurship strengthens both economic and social dimensions of poverty alleviation, demonstrating that targeted interventions can generate inclusive, sustainable benefits. ^109, 111^

Despite these positive outcomes, several gaps and contradictions remain in the literature. While water and sanitation initiatives, such as SHOFCO in Kenya and WaterHealth International in India and Africa, improve community access to safe drinking water and create micro-entrepreneurial opportunities, there is limited longitudinal evidence on the sustainability and long-term health and economic impacts of these programmes. ^113, 114^ Agricultural innovations, including Ethiopia’s adoption of drought-resistant seeds and Fairtrade cooperatives in Latin America, demonstrate enhanced productivity and market access, yet challenges persist in scalability, climate adaptation, and equitable benefit distribution. ^48, 116^ Comparative evaluation with previous studies indicates a consistent recognition of the role of resource-focused entrepreneurship in poverty reduction, but differences arise in reported effectiveness across regions due to variations in infrastructure, market integration, and policy support. ^118, 119^ Policy interventions should prioritise integrated approaches combining housing, energy, water, and agricultural support, backed by technical training and access to financing, to maximise sustainable livelihood outcomes. Future research should focus on longitudinal assessments of impact, cross-context replicability, and the interaction between different resource-based interventions to inform evidence-based strategies for resilient and inclusive development.

### Theoretical implications

The study examines relationship between literature review and the underpinning theoretical framework thereby identifying the persistent challenges and eminent gaps.

### Relationship between inclusive economic empowerment literature and diffusion of innovations theory

The literature on inclusive economic empowerment demonstrates a clear alignment with Rogers’ Diffusion of Innovations Theory, which emphasises the adoption of new ideas, practices, or technologies through social networks based on factors such as relative advantage, compatibility, complexity, trialability, and observability. ^29^ Social innovation in entrepreneurship, as evidenced by initiatives like Grameen Bank’s microfinance model in Bangladesh and M-Pesa mobile banking in Kenya, illustrates the diffusion process: these financial solutions offer a relative advantage by increasing income opportunities, are compatible with local socio-economic practices, and allow trialability through small-scale loans or digital banking usage. ^36, 37, 120^ Similarly, skills development programmes, including India’s National Skill Development Mission and Harambee Youth Employment Accelerator in South Africa, highlight how the observability of successful entrepreneurial outcomes encourages adoption among marginalised populations, promoting self-reliance and employability. ^39, 42^ These examples confirm that innovations that address pressing social and economic needs, when effectively communicated and adapted to local contexts, are more likely to diffuse widely and achieve systemic impact, consistent with the theoretical framework. ^30^

Despite this theoretical alignment, persistent challenges and gaps remain. Access to financial services, while transformative, does not guarantee long-term poverty reduction without complementary support such as mentorship, market linkages, and financial literacy. ^33, 35^ Similarly, vocational training and enterprise development programmes demonstrate variable long-term outcomes due to limited follow-up, context-specific barriers, and scalability constraints. ^40, 44^ The literature further reveals that socially driven enterprises, such as Fairtrade cooperatives in Latin America and Banapads in Uganda, achieve measurable benefits in employment and income, yet the diffusion of these models across different socio-cultural or infrastructural contexts remains underexplored. ^48, 50^ Policy interventions should, therefore, focus on integrating financial services with mentorship, skills training, and market access while fostering supportive regulatory and institutional environments that facilitate adoption of innovations. ^31, 32^ Future research should explore mechanisms to enhance the scalability, adaptability, and long-term sustainability of entrepreneurial innovations, examining how adoption rates can be maximised across diverse communities to achieve broader inclusive economic empowerment.

This examination demonstrates that while the Diffusion of Innovations Theory provides a robust framework for understanding the adoption of social entrepreneurial innovations, gaps exist in translating pilot successes into scalable, sustainable models that consistently reduce poverty and enhance inclusive development.

### Relationship between social inclusion and equity literature and diffusion of innovations theory

The literature on social inclusion and equity demonstrates a strong correspondence with Rogers’ Diffusion of Innovations Theory, which posits that innovations spread through social networks based on factors such as relative advantage, compatibility, complexity, trialability, and observability. ^29^ Gender-inclusive programmes, such as Rwanda’s Women’s Opportunity Centre and the SheTrades platform, illustrate the relative advantage of innovations that address entrenched gender inequalities while enhancing access to skills, resources, and markets. ^53, 55^ Similarly, youth-focused initiatives like the Tony Elumelu Foundation Entrepreneurship Programme and Youth Business International demonstrate compatibility and trialability, as young entrepreneurs can adopt new business practices, access mentorship, and secure financing, thereby facilitating observable benefits in employment creation and local economic stimulation. ^58, 60^ Support for vulnerable groups, exemplified by livelihood projects for refugees in Jordan and employment opportunities through Dialogue in the Dark, aligns with the theoretical framework by showing that socially adapted innovations can overcome adoption barriers, enhance participation, and foster financial independence. ^62^ Community-based decision-making models, such as SHOFCO in Kenya and participatory rural development projects in Bolivia, illustrate how observability and community engagement increase the likelihood of innovation adoption while ensuring context-specific relevance and equity. ^63, 66^

Despite this alignment, several persistent challenges and gaps emerge. While gender-inclusive and youth-focused programmes achieve measurable benefits, the diffusion of these innovations across broader populations is constrained by limited infrastructure, socio-cultural barriers, and resource availability. ^52, 59^ Longitudinal evidence on the sustained impact of these interventions on poverty reduction and social cohesion remains limited, particularly in contexts involving vulnerable groups with complex needs. ^4^ Comparative evaluation with previous studies suggests that the observable advantages of innovation may not always translate into equitable adoption, as differences in community readiness, institutional support, and access to capital influence uptake. ^54, 56^ Policy interventions should therefore prioritise creating enabling environments that integrate social entrepreneurship with skills development, inclusive financing, and participatory governance, while supporting the scaling of successful models. ^31, 32^ Future research should focus on understanding the mechanisms of adoption among diverse populations, evaluating long-term social and economic outcomes, and exploring strategies to enhance the replicability and sustainability of innovations that promote equity and social inclusion.

### Relationship between technological integration and digital innovation literature and diffusion of innovations theory

The literature on technological integration and digital innovation in entrepreneurship aligns closely with Rogers’ Diffusion of Innovations Theory, which explains how new ideas, practices, and technologies spread through social networks over time, influenced by relative advantage, compatibility, complexity, trialability, and observability. ^29^ Digital financial platforms, such as M-Pesa in Kenya and fintech solutions like Tala and Branch, exemplify the relative advantage and compatibility of innovations that address pressing financial inclusion gaps, enabling previously unbanked individuals to access credit, save securely, and engage in entrepreneurial activities. ^36, 71, 68^ E-learning and skills platforms, including Coursera for Refugees and IBM’s SkillsBuild initiative in India, illustrate how innovations that are observable, trialable, and compatible with local education and labour contexts enhance entrepreneurial capacity, employability, and pathways out of poverty. ^75, 76, 78^ Similarly, digital marketplaces such as Jumia, Etsy, and GoJek demonstrate how technological platforms can create scalable opportunities for marginalised producers, reflecting the theory’s principle that observability and trialability accelerate adoption when innovations produce tangible economic benefits. ^19, 80, 83^ Data-driven decision-making platforms, such as GiveDirectly and digital health initiatives in Latin America, align with the theoretical framework by ensuring innovations are relevant, context-specific, and measurable, which enhances trust and adoption among communities. ^82, 84, 86^ Collectively, these examples demonstrate that technological innovations in entrepreneurship can achieve systemic impact when they are effectively communicated, adapted to community needs, and observable in their benefits, in accordance with the Diffusion of Innovations Theory. ^30^

Despite these alignments, several persistent challenges and gaps are evident. The diffusion of digital innovations is often constrained by limited digital literacy, infrastructure deficiencies, and socio-economic disparities, which hinder equitable adoption among marginalised groups. ^73, 74^ While platforms such as M-Pesa and GoJek have achieved wide uptake, the long-term sustainability, scalability, and context-specific effectiveness of these models remain under-researched, particularly in rural or low-resource settings. ^70, 123^ E-learning programmes, although transformative, face challenges related to accessibility, internet connectivity, and integration with local labour market needs, limiting observable impact on employment and income generation. ^78, 122^ Policy interventions should therefore focus on promoting digital infrastructure, supporting digital literacy, and integrating technological innovations with complementary services such as mentorship, market access, and technical training to enhance adoption and sustainability. ^31, 68^ Future research should investigate long-term impacts, cross-context replicability, and strategies to reduce adoption barriers for vulnerable populations, while examining how digital innovations can be tailored to diverse socio-economic and cultural environments to maximise inclusive economic empowerment and sustainable development outcomes.

### Relationship between environmental sustainability and resilience literature and diffusion of innovations theory

The literature on environmental sustainability and resilience in entrepreneurship demonstrates a strong alignment with Rogers’ Diffusion of Innovations Theory, which explains the adoption of innovations through social networks based on relative advantage, compatibility, complexity, trialability, and observability. ^29^ Green business models, such as SELCO’s solar-powered lighting solutions in India and Waste Concern’s composting enterprise in Bangladesh, exemplify innovations with clear relative advantage and observable benefits, as they simultaneously generate income and reduce environmental harm. ^25, 72, 88^ Climate-resilient livelihood initiatives, including Ethiopia’s drought-resistant crops and Green Antz in the Philippines, reflect compatibility and trialability, as communities can adopt context-specific sustainable practices that safeguard income while mitigating climate risks. ^89– 91^ Renewable energy entrepreneurship, illustrated by Zola Electric in Tanzania and community-based hydropower projects in Nepal, demonstrates observability, as the tangible benefits of affordable energy access, cost reduction, and enhanced productive activities promote adoption and replication. ^95– 97^ Circular economy solutions, such as Conceptos Plásticos in Colombia and Trashy Bags Africa in Ghana, further exemplify how innovative waste-to-resource models combine social and economic impact with environmental sustainability, reinforcing the adoption of scalable and socially desirable practices. ^100– 102^ Collectively, these examples illustrate how socially innovative entrepreneurial ventures align with the theoretical principles of diffusion, enhancing the likelihood of adoption and widespread impact.

Despite these positive alignments, several persistent challenges and gaps are evident. First, the scalability and replication of environmentally sustainable innovations are constrained by limited access to financial resources, technological infrastructure, and market linkages, particularly in low-income or rural settings. ^25, 87^ Second, while the observable benefits of renewable energy and circular economy initiatives are clear, there is limited longitudinal evidence on their sustained economic, environmental, and social impact, making it difficult to evaluate long-term efficacy. ^95, 99^ Third, complex socio-cultural and institutional barriers may slow the adoption of green and climate-resilient practices, highlighting the need for tailored engagement strategies that account for local norms and capacities. ^90, 91^ Policy interventions should therefore focus on supporting access to green finance, facilitating technology transfer, incentivising sustainable business models, and integrating environmental entrepreneurship into national development strategies. ^3, 31^ Areas for further research include examining long-term impacts of sustainable entrepreneurship on poverty reduction, exploring effective mechanisms for scaling environmentally innovative ventures across diverse contexts, and investigating how socio-cultural factors influence adoption and diffusion of green innovations in vulnerable communities.

### Relationship between sustainable livelihoods and resource access literature and diffusion of innovations theory

The literature on sustainable livelihoods and resource access in entrepreneurship strongly aligns with Rogers’ Diffusion of Innovations Theory, which posits that innovations spread through social networks based on factors such as relative advantage, compatibility, complexity, trialability, and observability. ^29^ Affordable housing initiatives, such as Conceptos Plásticos in Colombia and India’s Incremental Housing model through SPARC, demonstrate relative advantage and observability, as communities gain immediate improvements in living conditions while participating in construction processes. ^106– 108^ Renewable energy innovations, including Zola Electric in Tanzania and micro-hydropower projects in Nepal, illustrate compatibility and trialability, allowing households to adopt solutions that reduce energy costs, enable productive activities, and foster income generation. ^76, 95, 96, 111^ Water and sanitation initiatives, such as SHOFCO in Kenya and WaterHealth International in rural India and Africa, provide observable benefits that are culturally adaptable and economically advantageous, demonstrating how accessible resource innovations can diffuse effectively through local communities. ^113– 115^ Agricultural innovations, including Ethiopia’s adoption of drought-resistant seeds, Fairtrade-certified cooperatives in Latin America, and Digital Green’s video-based training in India, highlight the theory’s applicability by showcasing innovations that are practical, compatible with local farming practices, and demonstrably beneficial to income and food security. ^48, 117, 119^ These examples confirm that socially innovative entrepreneurial ventures addressing basic resource needs embody the principles of diffusion and have the potential for widespread adoption, leading to systemic improvements in poverty reduction and sustainable development.

Despite the alignment with Diffusion of Innovations Theory, several challenges and gaps persist. Scalability of housing, energy, water, and agricultural innovations remains constrained by limited financial resources, technological infrastructure, and institutional support, which can hinder adoption in resource-poor communities. ^103, 109^ Longitudinal data on the sustained economic and social impact of these initiatives are limited, reducing the ability to assess effectiveness over time. ^5, 116^ Additionally, socio-cultural and policy barriers may restrict adoption and replication across diverse contexts, particularly in areas with weak governance or limited community engagement. ^104, 112^ Policy interventions should prioritise integrating resource-access initiatives with financial support, technical assistance, and capacity-building programmes, while encouraging participatory approaches that enhance adoption rates and sustainability. ^1, 31^ Further research is needed to evaluate the long-term impacts of resource-based innovations, investigate strategies for scaling solutions across heterogeneous contexts, and explore how social, cultural, and institutional factors influence the diffusion and sustainability of entrepreneurial interventions targeting poverty reduction.

### Practical implications

The practical implications of this study, highlight actionable strategies for policymakers, development practitioners, and entrepreneurial actors seeking to reduce poverty and promote sustainable development through social innovation.

In the realm of Inclusive Economic Empowerment, the study underscores the importance of providing marginalised populations with access to financial services, vocational training, and enterprise support. Microfinance schemes, mobile banking platforms, and mentorship programmes not only increase income-generating opportunities but also empower individuals to scale small businesses sustainably. ^37, 39, 44, 120^ Practically, this suggests that governments and development agencies should implement policies that integrate financial inclusion with skills development and business support, thereby creating an enabling ecosystem that facilitates entrepreneurship for poverty reduction.

Regarding Social Inclusion and Equity, the findings demonstrate that targeted programmes for women, youth, refugees, and persons with disabilities enhance participation in economic activities, dismantle structural inequalities, and promote social cohesion. ^53, 57, 63^ This implies that practical interventions should prioritise inclusive policy frameworks, tailored training, and cooperative networks that ensure vulnerable groups have equitable access to entrepreneurial opportunities, enabling their meaningful integration into local and global markets.

The theme of Technological Integration and Digital Innovation reveals the transformative potential of digital financial services, e-learning platforms, and data-driven approaches in fostering entrepreneurship and sustainable livelihoods. ^68, 75, 83^ Practically, this indicates that policymakers and business leaders should invest in digital infrastructure, provide affordable access to fintech and e-commerce platforms, and support training in digital literacy and entrepreneurial skills. Such measures can accelerate the adoption of innovations, expand market access for marginalised producers, and enhance the scalability of socially impactful enterprises.

For Environmental Sustainability and Resilience, the study highlights how green business models, renewable energy initiatives, climate-resilient livelihoods, and circular economy solutions can simultaneously reduce poverty and protect ecosystems. ^11, 87, 89, 93^ The practical implication is that governments, NGOs, and entrepreneurs should integrate environmental considerations into social enterprise strategies, encourage the adoption of eco-friendly technologies, and provide incentives for businesses that create both social and environmental value. This approach strengthens community resilience against climate shocks while promoting sustainable income generation.

Finally, in terms of Sustainable Livelihoods and Resource Access, the research shows that providing access to essential resources such as affordable housing, clean energy, water, and improved agricultural technologies empowers communities and fosters long-term economic resilience. ^117^ Practically, development practitioners should design integrated interventions that address multiple dimensions of resource access while linking them with income-generating activities. This ensures that vulnerable populations not only survive but thrive, achieving both immediate and long-term improvements in well-being.

Collectively, these implications suggest that a holistic, multi-sectoral approach combining financial, social, technological, environmental, and resource-focused interventions is essential for translating social innovation in entrepreneurship into tangible poverty reduction and sustainable development outcomes. By addressing both structural and functional barriers, stakeholders can foster inclusive growth and systemic change that benefits the most marginalised communities.

### Direction for future research

Based on this review several recommendations for future research emerge to address persistent gaps and enhance understanding of how social innovation in entrepreneurship drives poverty reduction.

Firstly, longitudinal studies are needed to assess the sustained impact of social innovation on marginalized populations. While initiatives such as microfinance, mobile banking, vocational training, and youth entrepreneurship programmes have demonstrated short-term benefits in income generation and employment, there is limited evidence on long-term business survival, household welfare, and systemic poverty alleviation. ^39, 57, 120^ Future research should track beneficiaries over extended periods to determine the durability of economic empowerment and the mechanisms through which entrepreneurial interventions produce lasting change.

Secondly, there is a need for comparative cross-context analyses to understand how socio-cultural, economic, and regulatory environments influence the adoption and effectiveness of social innovations. Studies on gender-inclusive programmes, refugee-focused initiatives, and participatory community models have shown promising outcomes in specific countries, but the scalability and transferability of these interventions across different regions remain underexplored. ^53, 68^ Comparative research can illuminate context-specific enablers and barriers, providing policymakers and practitioners with evidence to tailor interventions effectively.

Thirdly, technology-focused research should investigate the long-term efficacy of digital innovations, including e-learning, fintech platforms, and data-driven decision-making, in enhancing entrepreneurship and reducing poverty. ^68, 75, 83^ Future studies could examine the intersection of digital literacy, infrastructure availability, and behavioural adoption, identifying strategies to increase uptake among marginalized populations. Additionally, understanding potential unintended consequences, such as digital exclusion or inequities in access, is critical to designing inclusive technology interventions.

Fourthly, research on environmental sustainability and resilience requires deeper exploration of the interactions between entrepreneurial activities and climate adaptation. While green business models, renewable energy initiatives, and circular economy solutions demonstrate dual social and environmental benefits, there is limited evidence on the long-term economic viability and environmental impact of these ventures. ^87, 89, 100^ Future studies should adopt integrated evaluation frameworks that measure social, economic, and ecological outcomes simultaneously, providing comprehensive insights into sustainable development pathways.

Finally, future research should explore integrated, multi-dimensional approaches that combine resource access, income generation, social inclusion, and environmental sustainability. Existing studies often focus on single interventions, such as housing, water, energy, or agriculture, without fully capturing their interconnected effects on poverty reduction and community resilience. ^62^ Investigating the synergies and trade-offs among multiple interventions can guide the design of holistic programmes that maximise social impact and support systemic change.

### Policy recommendations

Based on the review, several concrete policy recommendations emerge to enhance poverty reduction through social innovation in entrepreneurship, targeting sustainable development outcomes.

Governments should promote gender-inclusive entrepreneurship by implementing policies that provide targeted financial support, training, and market access for women entrepreneurs. For example, Rwanda’s Women’s Opportunity Centre and the SheTrades initiative have successfully empowered women with skills and cooperative networks, enabling access to international markets and reducing gender inequality. ^53, 55^ Policymakers, such as ministries of trade and women’s affairs, can collaborate with international organisations and NGOs to scale such programmes, while monitoring and evaluating long-term impacts on income generation and social inclusion.

To strengthen youth entrepreneurship, policies should incentivise mentorship programmes, seed funding, and vocational training for young innovators. The Tony Elumelu Foundation in Nigeria and Youth Business International in Europe illustrate how structured support can generate sustainable businesses and reduce youth unemployment. ^58, 60^ National education ministries and labour departments can partner with private sector actors and development agencies to integrate entrepreneurship education into formal curricula and provide incubator support for start-ups, fostering economic inclusion and social cohesion.

Support for vulnerable groups should be embedded in national entrepreneurship strategies. Social enterprises in Jordan and initiatives like Dialogue in the Dark demonstrate that entrepreneurship can restore dignity and financial independence for displaced refugees and persons with disabilities. ^62^ Governments can collaborate with humanitarian agencies, disability advocacy groups, and local NGOs to design targeted livelihood programmes, offering training, accessible financing, and employment opportunities that ensure equity and social protection.

Policymakers should also leverage technological integration to enhance financial inclusion, market access, and skills development. Mobile banking platforms such as M-Pesa in Kenya and fintech solutions like Tala have expanded credit access and entrepreneurial opportunities for underserved populations. ^37, 71^ National financial regulators, technology ministries, and fintech companies can develop policies that promote affordable digital infrastructure, financial literacy, and e-learning platforms, ensuring marginalized communities can participate in innovation-driven sectors.

To promote environmental sustainability and resilience, governments should incentivise green entrepreneurship through grants, tax breaks, and technical assistance for renewable energy, circular economy, and climate-resilient agriculture initiatives. Enterprises like SELCO in India, Zola Electric in Tanzania, and Conceptos Plásticos in Colombia illustrate how environmentally sustainable businesses can generate income while addressing social and ecological challenges. ^25, 95, 100^ Collaboration among ministries of environment, energy, and agriculture, alongside development banks and private investors, can create an enabling environment for scaling green enterprises, ensuring both economic and ecological benefits.

Finally, policies should facilitate integrated, community-driven approaches that combine housing, energy, water, and agriculture to foster sustainable livelihoods. Initiatives such as SPARC in India, SHOFCO in Kenya, and Fairtrade cooperatives in Latin America demonstrate the effectiveness of participatory, multi-sectoral interventions that address basic needs while generating income. ^48, 107, 113^ Governments, local authorities, community organisations, and international agencies can co-develop frameworks that empower communities to co-create solutions, enhancing local ownership, resilience, and inclusive economic growth.

## Conclusion

This review demonstrates that social innovation in entrepreneurship is a powerful mechanism for poverty reduction and sustainable development. By integrating social objectives with entrepreneurial initiatives, marginalized populations, including women, youth, refugees, and persons with disabilities, gain access to economic opportunities that were previously out of reach. Socially innovative enterprises not only provide income-generating activities but also foster social cohesion, empowerment, and resilience, illustrating the multidimensional impact of entrepreneurship beyond mere financial gains.

The practical implications of these findings highlight the importance of designing entrepreneurship programmes that are inclusive, technology-driven, environmentally conscious, and aligned with community needs. Policies and interventions that support gender equality, youth engagement, skill development, financial inclusion, renewable energy access, and participatory approaches have the potential to create sustainable livelihoods while simultaneously addressing systemic social and environmental challenges. These initiatives underscore the need for collaboration among governments, non-governmental organisations, private sector actors, and local communities to ensure that innovations are widely adopted and their benefits equitably distributed.

From a personal perspective, the integration of social innovation into entrepreneurship presents a transformative pathway for addressing poverty in a holistic manner. The convergence of economic empowerment, social equity, technological advancement, and environmental stewardship offers a blueprint for sustainable development that is both scalable and adaptable to diverse contexts. However, persistent challenges remain, including ensuring long-term sustainability, scaling successful models, and measuring tangible impact on marginalized populations. Addressing these gaps requires ongoing research, policy refinement, and commitment from all stakeholders to foster an ecosystem where socially innovative entrepreneurship can thrive. In essence, social innovation in entrepreneurship is not only a tool for economic growth but a catalyst for building inclusive, resilient, and sustainable societies.

### Limitations of the study

The study has two main limitations. First, it relies primarily on secondary literature and illustrative examples, which limits the generalisability of the findings across diverse socio-economic and cultural contexts. Second, there is a lack of empirical and longitudinal data to assess the long-term sustainability, scalability, and measurable impact of socially innovative entrepreneurial initiatives.

## Data Availability

There is no underlying data associated with this review. Ongesa, T. (2025) *Social Innovation in Entrepreneurship: A Strategic Pathway to Poverty Reduction for Sustainable Development: A systematic Literature Review.* Zenodo. https://doi.org/10.5281/zenodo.17937306 ^124^ **PRISMA checklist and flowchart:** Zenodo. https://doi.org/10.5281/zenodo.17937306 ^124^ Data are available under the terms of the Creative Commons Attribution 4.0 International license (CC-BY 4.0).
