# Supplementary material for: Social Innovation in Entrepreneurship: A Strategic Pathway to Poverty Reduction for Sustainable Development: A systematic Literature Review
Source: F1000Res. 2026 Apr 27;14:1464. Originally published 2025 Dec 27. [Version 2] doi: 10.12688/f1000research.174615.2 (PMC13294669; doi:10.12688/f1000research.174615.2)
Supplement: Peer review history

# Reviewer report: Reviewer response for version 2

**DOI:** 10.5256/f1000research.198875.r494455

**Published:** 2026-06-24

**Authors:** Dvorský Ján

**Affiliations:** 1 University of Zilina, Žilina, Slovakia

+ The article is engaging and presents insightful findings that enrich both theory and practice.

 + I recommend that the author review the empirical study from Ghana, *'Ref 1 - (*Randy Afful C., et al, 2026) *'*, as it can significantly enrich the theoretical and practical understanding of the issues addressed (section practical implication - governments).

 + Briefly, I recommend outlining potential avenues for future research in the final version of the manuscript.

Are the rationale for, and objectives of, the Systematic Review clearly stated?

Yes

Is the statistical analysis and its interpretation appropriate?

Not applicable

If this is a Living Systematic Review, is the ‘living’ method appropriate and is the search schedule clearly defined and justified? (‘Living Systematic Review’ or a variation of this term should be included in the title.)

Yes

Are sufficient details of the methods and analysis provided to allow replication by others?

Yes

Are the conclusions drawn adequately supported by the results presented in the review?

Yes

Reviewer Expertise:

CSR, ESG, SMEs, AI, small businesses, HRM, business environment

I confirm that I have read this submission and believe that I have an appropriate level of expertise to confirm that it is of an acceptable scientific standard.

## References

[1] : Evaluating the Impact of Strategic Sustainable Orientation and Socioeconomic Policies on the Mining Sector in Ghana. *Journal of Business Sectors* .2026;4(1) : 10.62222/YEBO6947 43-56 10.62222/YEBO6947

# Reviewer report: Reviewer response for version 2

**DOI:** 10.5256/f1000research.198875.r487488

**Published:** 2026-06-09

**Authors:** Thadathibesra Phuthong

**Affiliations:** 1 Silpakorn University, Phetchaburi, Thailand

Thank you for the paper submission. Some comments will help to improve it.

 The author has made notable revisions that significantly enhance the manuscript, including harmonizing the database set, adding Boolean search strings, providing justifications for the inclusion of Google Scholar, refining PRISMA screening procedures, and offering concrete policy recommendations. However, several critical concerns remain unresolved, including inconsistencies in database reporting: the Methods section lists five databases, whereas the Results section indicates only three were used. Additionally, the count of included studies is inconsistent: the narrative claims 12 studies were included, whereas the PRISMA flow diagram shows 15, creating confusion about the evidence base. The manuscript lacks engagement with issues such as heterogeneity, reporting bias, and the certainty of the evidence, despite references in the PRISMA checklist.

 Further, the boundary between systematically included evidence and illustrative narrative has not been addressed, obscuring which claims are derived from included studies. The treatment of Diffusion of Innovations Theory, while acknowledging its limited operationalization, still includes elements that reviewers have criticized, leading to tension in the manuscript.

 Several reviewer points show little engagement, including criticisms of the evidence base's geographical representation and claims of empowerment that lack data support. The tone remains overly promotional, with a personal anecdote included that is inappropriate for a systematic review. Issues concerning reproducibility are evident, as the claim of an open workflow conflicts with a lack of transparency in data availability.

 Furthermore, credibility is called into question by the description of independent reviewers when only a single author is mentioned. Lastly, the structural arrangement of sections creates repetitiveness, and consolidating themes would enhance clarity and brevity. In conclusion, while the revision has improved several aspects, critical methodological and analytical issues remain unaddressed, necessitating a recommendation for Major Revision to meet systematic-review standards.

Are the rationale for, and objectives of, the Systematic Review clearly stated?

Yes

Is the statistical analysis and its interpretation appropriate?

Yes

If this is a Living Systematic Review, is the ‘living’ method appropriate and is the search schedule clearly defined and justified? (‘Living Systematic Review’ or a variation of this term should be included in the title.)

Yes

Are sufficient details of the methods and analysis provided to allow replication by others?

No

Are the conclusions drawn adequately supported by the results presented in the review?

No

Reviewer Expertise:

Sustainable Development, Entrepreneurship

I confirm that I have read this submission and believe that I have an appropriate level of expertise to confirm that it is of an acceptable scientific standard, however I have significant reservations, as outlined above.

# Reviewer report: Reviewer response for version 1

**DOI:** 10.5256/f1000research.192533.r457474

**Published:** 2026-02-25

**Authors:** Junaid Aftab

**Affiliations:** 1 Tongji University, Shanghai, China

pubThank you for inviting me to review the manuscript "Social Innovation in Entrepreneurship: A Strategic Pathway to Poverty Reduction for Sustainable Development: A Systematic Literature Review." My comments are below

 This manuscript addresses the linkage between social innovation, entrepreneurship, poverty reduction, and sustainable development. However, substantial conceptual, methodological, and analytical weaknesses limit its contribution in its current form.

 First, the central claim of novelty is overstated. The manuscript asserts that limited attention has been given to the link between entrepreneurship and social innovation in poverty reduction. This is not sufficiently substantiated. The fields of social entrepreneurship, inclusive innovation, and sustainable entrepreneurship are already well established. The review does not clearly demonstrate how it advances theory, methods, or evidence beyond existing systematic and narrative reviews.

 The manuscript would benefit from a dedicated literature review section that situates the study within prior work and clarifies the research gap.

 Second, the methodological rigor does not match the PRISMA framing. Although the review claims PRISMA 2020 compliance, key elements are insufficiently reported. The search strategy lacks fully reproducible strings. Database coverage is inconsistent across sections. The inclusion of Google Scholar without clear screening boundaries raises concerns about reproducibility. The decision to restrict to English-only publications introduces potential bias without justification. The exclusion of grey literature is problematic in a field where many high-quality impact evaluations are published by development agencies.

 Third, the synthesis strategy is weak. Only twelve studies were included out of 1,284 identified records. This extremely narrow evidence base raises questions about either the search strategy or the inclusion criteria. The manuscript states that vote counting by direction of effect was used for quantitative synthesis. This approach is methodologically limited and prone to misleading inferences, particularly without standardized effect sizes or confidence intervals. There is no serious engagement with heterogeneity, publication bias, or certainty of evidence despite listing these items in the PRISMA checklist table.

 Fourth, the results section blends systematic review findings with illustrative case examples drawn from broader literature. Many of the named initiatives function as descriptive examples rather than part of the final included twelve studies. The boundary between included empirical evidence and general background literature is unclear.

 Fifth, the thematic structure reads as a narrative literature review. Sections on inclusive economic empowerment, social inclusion, digital innovation, environmental sustainability, and sustainable livelihoods are largely descriptive. The analysis repeats claims of positive impacts with limited critical interrogation of study quality, magnitude of effects, or contextual trade-offs. Contradictory evidence is acknowledged briefly but not deeply examined.

 Sixth, the application of Diffusion of Innovations Theory is conceptually thin. The theory is described and then retrofitted to diverse cases. There is no systematic operationalization of constructs such as relative advantage or observability in the included studies.

 Seventh, the manuscript over-relies on widely cited examples such as Grameen Bank, M-Pesa, SELCO, and Fairtrade cooperatives. The review would benefit from deeper engagement with less prominent, rigorously evaluated interventions drawn from the final twelve studies.

 Eighth, the equity lens is insufficiently developed. Gender, youth, disability, and rural contexts are mentioned, but no systematic comparison of differential impacts across groups is presented.

 Ninth, the discussion of limitations is not sufficiently critical. The manuscript should acknowledge the small sample size, possible selection bias, language bias, sectoral concentration, and limited generalizability, as well as the constraints of vote counting and narrative thematic coding.

 Tenth, the policy recommendations remain general. Stronger policy relevance would require linking specific findings from the included studies to concrete institutional design implications.

 In terms of writing, the manuscript is organized but repetitive. Several sections reiterate similar arguments with minor variation. The conceptual frameworks presented in multiple figures add limited analytical value.

  To meet systematic review standards, the author must strengthen methodological transparency, clearly separate included evidence from illustrative examples, deepen analytical synthesis, and improve theoretical integration. A dedicated literature review section is needed to position the study relative to prior research and clarify the contribution. Substantial revision is required before the manuscript can make a scholarly contribution.

 Thank you for inviting me to review the manuscript "Social Innovation in Entrepreneurship: A Strategic Pathway to Poverty Reduction for Sustainable Development: A Systematic Literature Review." My comments are below

 This manuscript addresses the linkage between social innovation, entrepreneurship, poverty reduction, and sustainable development. However, substantial conceptual, methodological, and analytical weaknesses limit its contribution in its current form.

 First, the central claim of novelty is overstated. The manuscript asserts that limited attention has been given to the link between entrepreneurship and social innovation in poverty reduction. This is not sufficiently substantiated. The fields of social entrepreneurship, inclusive innovation, and sustainable entrepreneurship are already well established. The review does not clearly demonstrate how it advances theory, methods, or evidence beyond existing systematic and narrative reviews.

 The manuscript would benefit from a dedicated literature review section that situates the study within prior work and clarifies the research gap.

 Second, the methodological rigor does not match the PRISMA framing. Although the review claims PRISMA 2020 compliance, key elements are insufficiently reported. The search strategy lacks full reproducible strings. Database coverage is inconsistent across sections. The inclusion of Google Scholar without clear screening boundaries raises concerns about reproducibility. The decision to restrict to English-only publications introduces potential bias without justification. The exclusion of grey literature is problematic in a field where many high-quality impact evaluations are published by development agencies.

 Third, the synthesis strategy is weak. Only twelve studies were included out of 1,284 identified records. This extremely narrow evidence base raises questions about either the search strategy or the inclusion criteria. The manuscript states that vote counting by direction of effect was used for quantitative synthesis. This approach is methodologically limited and prone to misleading inferences, particularly without standardized effect sizes or confidence intervals. There is no serious engagement with heterogeneity, publication bias, or certainty of evidence despite listing these items in the PRISMA checklist table.

 Fourth, the results section blends systematic review findings with illustrative case examples drawn from broader literature. Many of the named initiatives function as descriptive examples rather than part of the final included twelve studies. The boundary between included empirical evidence and general background literature is unclear.

 Fifth, the thematic structure reads as a narrative literature review. Sections on inclusive economic empowerment, social inclusion, digital innovation, environmental sustainability, and sustainable livelihoods are largely descriptive. The analysis repeats claims of positive impacts with limited critical interrogation of study quality, magnitude of effects, or contextual trade-offs. Contradictory evidence is acknowledged briefly but not deeply examined.

 Sixth, the application of Diffusion of Innovations Theory is conceptually thin. The theory is described and then retrofitted to diverse cases. There is no systematic operationalization of constructs such as relative advantage or observability in the included studies.

 Seventh, the manuscript over-relies on widely cited examples such as Grameen Bank, M-Pesa, SELCO, and Fairtrade cooperatives. The review would benefit from deeper engagement with less prominent, rigorously evaluated interventions drawn from the final twelve studies.

 Eighth, the equity lens is insufficiently developed. Gender, youth, disability, and rural contexts are mentioned, but no systematic comparison of differential impacts across groups is presented.

 Ninth, the discussion of limitations is not sufficiently critical. The manuscript should acknowledge the small sample size, possible selection bias, language bias, sectoral concentration, and limited generalizability, as well as the constraints of vote counting and narrative thematic coding.

 Tenth, the policy recommendations remain general. Stronger policy relevance would require linking specific findings from the included studies to concrete institutional design implications.

 In terms of writing, the manuscript is organized but repetitive. Several sections reiterate similar arguments with minor variation. The conceptual frameworks presented in multiple figures add limited analytical value.

  To meet systematic review standards, the author must strengthen methodological transparency, clearly separate included evidence from illustrative examples, deepen analytical synthesis, and improve theoretical integration. A dedicated literature review section is needed to position the study relative to prior research and clarify the contribution. Substantial revision is required before the manuscript can make a scholarly contribution.

Are the rationale for, and objectives of, the Systematic Review clearly stated?

Partly

Is the statistical analysis and its interpretation appropriate?

Partly

If this is a Living Systematic Review, is the ‘living’ method appropriate and is the search schedule clearly defined and justified? (‘Living Systematic Review’ or a variation of this term should be included in the title.)

Yes

Are sufficient details of the methods and analysis provided to allow replication by others?

No

Are the conclusions drawn adequately supported by the results presented in the review?

Partly

Reviewer Expertise:

Entrepreneurship; CSR; Sustainability

I confirm that I have read this submission and believe that I have an appropriate level of expertise to state that I do not consider it to be of an acceptable scientific standard, for reasons outlined above.

# Reviewer report: Reviewer response for version 1

**DOI:** 10.5256/f1000research.192533.r446371

**Published:** 2026-02-17

**Authors:** Ayyoob Arangodan

**Affiliations:** 1 University of Calicut, Thenhipalam, Kerala, India

The manuscript addresses an important and timely topic and demonstrates strong familiarity with the social innovation literature. However, incorporating the following changes would significantly strengthen the paper.

1. The review claims to synthesise “the last decade of SIE scholarship” and provide policy-relevant conclusions, yet only 12 studies (approximately 1% of identified records) were included. This severely limits the strength and generalisability of the conclusions and should be more explicitly acknowledged as a constraint on inference.
2. High-profile examples (e.g., M-Pesa, Grameen Bank, SELCO) are repeatedly cited, even when they are not part of the 12 included studies, which blurs the boundary between systematic evidence and narrative illustration.
3. Although equity (gender, youth, and disability) is emphasised, only 4 of the 12 included studies explicitly measure empowerment or capabilities, limiting the evidentiary basis for strong equity-related conclusions.
4. Different sections report different databases (Scopus, Google Scholar, JSTOR vs. Scopus, Web of Science, ABI/INFORM). This inconsistency raises concerns about transparency and reproducibility of the search strategy. A single, harmonised list should be provided.
5. The inclusion of Google Scholar, without a clear screening or deduplication protocol, risks introducing selection bias. The authors should justify its use and explain how quality control was maintained.
6. Keywords are listed, but full Boolean strings, field tags, and database-specific adaptations are missing, which prevents replication in line with PRISMA 2020 standards.
7. Although a PRISMA checklist is included, key items (selection process reviewers, disagreement resolution, and automation tools) are described superficially rather than operationally.
8. While Diffusion of Innovations Theory is repeatedly referenced, it is not systematically operationalised in study selection, coding, or synthesis. The theory functions more as a framing device than an analytical tool.
9. One of the stated aims is cross-context comparison, yet the included studies are heavily skewed toward Sub-Saharan Africa and South Asia, with minimal representation of advanced economies.
10. The review restricts inclusion to 2019–2025 yet draws strong conclusions about long-term sustainability and systemic change, which cannot be robustly supported within such a short evidence window.
11. Recommendations are broad and aspirational but lack clear prioritisation, cost considerations, or feasibility constraints, limiting their usefulness for policymakers and practitioners.
12. Some sections adopt normative or promotional language (“demonstrates vital importance,” “proves transformative”), which is not fully supported by the limited and heterogeneous evidence base. A more cautious tone is recommended.

Are the rationale for, and objectives of, the Systematic Review clearly stated?

Partly

Is the statistical analysis and its interpretation appropriate?

Partly

If this is a Living Systematic Review, is the ‘living’ method appropriate and is the search schedule clearly defined and justified? (‘Living Systematic Review’ or a variation of this term should be included in the title.)

Not applicable

Are sufficient details of the methods and analysis provided to allow replication by others?

Partly

Are the conclusions drawn adequately supported by the results presented in the review?

Partly

Reviewer Expertise:

CSR, ESG, SDG 12, Corporate Resilience, Sustainability, Social Entrepreneurship

I confirm that I have read this submission and believe that I have an appropriate level of expertise to confirm that it is of an acceptable scientific standard, however I have significant reservations, as outlined above.
